# TB-IECS: an accurate machine learning-based scoring function for virtual screening

**DOI:** 10.1186/s13321-023-00731-x

**Published:** 2023-07-04

**Authors:** Xujun Zhang, Chao Shen, Dejun Jiang, Jintu Zhang, Qing Ye, Lei Xu, Tingjun Hou, Peichen Pan, Yu Kang

**Affiliations:** 1 Innovation Institute for Artificial Intelligence in Medicine of, Zhejiang University, College of Pharmaceutical Sciences, Zhejiang University, Hangzhou, 310058 Zhejiang China; 2 Institute of Bioinformatics and Medical Engineering, School of Electrical and Information Engineering, Jiangsu University of Technology, Changzhou, 213001 China

**Keywords:** Scoring function, Machine learning, Virtual screening, Theory-based interaction energy component

## Abstract

Machine learning-based scoring functions (MLSFs) have shown potential for improving virtual screening capabilities over classical scoring functions (SFs). Due to the high computational cost in the process of feature generation, the numbers of descriptors used in MLSFs and the characterization of protein–ligand interactions are always limited, which may affect the overall accuracy and efficiency. Here, we propose a new SF called TB-IECS (theory-based interaction energy component score), which combines energy terms from Smina and NNScore version 2, and utilizes the eXtreme Gradient Boosting (XGBoost) algorithm for model training. In this study, the energy terms decomposed from 15 traditional SFs were firstly categorized based on their formulas and physicochemical principles, and 324 feature combinations were generated accordingly. Five best feature combinations were selected for further evaluation of the model performance in regard to the selection of feature vectors with various length, interaction types and ML algorithms. The virtual screening power of TB-IECS was assessed on the datasets of DUD-E and LIT-PCBA, as well as seven target-specific datasets from the ChemDiv database. The results showed that TB-IECS outperformed classical SFs including Glide SP and Dock, and effectively balanced the efficiency and accuracy for practical virtual screening.

**Supplementary Information:**

The online version contains supplementary material available at 10.1186/s13321-023-00731-x.

## Introduction

Structure-based virtual screening (SBVS) has become one of the common approaches for drug discovery. As a core technology widely used in SBVS, molecular docking can predict the binding modes of protein–ligand complexes and estimate the binding affinities using scoring functions (SFs). A number of docking programs, such as Surflex, AutoDock, Glide, and Gold, have been developed and widely used by pharmaceutical companies and research institutions [1–4] The reliability of docking programs largely depends on the quality of conformational sampling methods and SFs. Unfortunately, most SFs implemented in the docking programs are calculated simply by multiple linear regression, which in many cases leads to insufficient accuracy [5, 6].

Classical SFs can be roughly divided into three categories: force-field based, knowledge-based, and empirical-based. Linear additive feature is one of the simplest algorithms and is often utilized to characterize protein–ligand interactions in these models [7, 8] However, linear SFs are incapable to capture the accurate description of the various binding patterns of ligands and may be less effective in large-scale VS applications [9] With the rapid increasing of computational capacity and the explosive growth of available protein–ligand structures and binding affinity data, MLSFs based on a wide range of descriptors generated from protein–ligand complexes have been developed, and some of them exhibit better performance over traditional SFs [5, 6, 9–16] To construct a reliable MLSF, three components are required: (1) an appropriate ML algorithm for classification and/or regression analysis, (2) a good representation of protein–ligand interactions, and (3) an authoritative benchmark for constructing and testing models. Support vector machine (SVM), random forest (RF), gradient boosting decision tree (GBDT) and artificial neural network (ANN) are the most frequently used ML algorithms in MLSFs. For instance, RF-Score constructed by Pedro J. et al [17] could increase considerably the accuracy in certain scenarios. In recent years, deep learning (DL) algorithms, including graph neural network (GNN) and convolutional neural network (CNN), have drawn increasing attention in MLSF study [7, 18–22] Within the CNN-based SFs, KDEEP and RosENet have demonstrated significant effectiveness, while InteractionGraphNet, PIGNet, and RTMScore exemplify the performance of GNN-based SFs [18, 20, 23–25] Each of these models offers a high level of accuracy in predicting binding affinity. DL algorithms hunger for large scale high-quality datasets, but the available experimentally determined data are often limited, which makes it difficult to develop an accurate and robust model. Hence, traditional ML algorithms such as RF and GBDT are still regarded as powerful tools in MLSF development. From the perspective of descriptors, SFs can be improved by developing more descriptors to comprehensively capture key protein–ligand interactions. A common approach to extract the features from protein–ligand complexes is to decompose existing traditional SFs into individual energy terms [26] For example, Ashtawy et al. developed two models named BgN-Score and BsN-Score using the descriptors extracted from X-score, AffiScore, GOLD, and RF-score [27] Li et al*.* proposed a SF called XGB-Score based on the eXtreme Gradient Boosting (XGBoost) algorithm using the energy terms from RF-Score and Vina [28] However, it remains unclear whether the regrouped energy terms with redundant features could successfully capture protein–ligand interactions.

In this study, we proposed a new SF, named theory-based interaction energy component score (TB-IECS), developed by the classical XGBoost algorithm based on the energy terms obtained from Smina and NNScore 2. During the modeling process, we systematically explored the impact of different feature combinations, ML algorithms and feature vector lengths on model performance. Besides, the screening power of the models was assessed based on the DUD-E and LIT-PCBA datasets [29, 30] In order to explore the screening power of TB-IECS, seven TB-IECS models were trained for seven different drug targets and utilized to screen the ChemDiv database. In this study, we aimed to clarify the following questions: (1) Can feature combinations based on formula representation and physicochemical properties improve the predictive performance? (2) How do features affect model accuracy and efficiency? (3) Can TB-IECS be applied to VS?

## Materials and methods

### Dataset collection

The benchmarks used in this study are the diverse subset of DUD-E (Dataset I) and a subset of LIT-PCBA (Dataset II). DUD-E contains 22,886 ligands with the experimental activity data against 102 targets. For each active compound, 50 decoys with similar physicochemical properties but dissimilar 2-D topology are provided. The diverse set was provided by the DUD-E database, where 112,796 ligands associated with eight targets from different protein families were included.

Considering the hidden bias in DUD-E as observed by numerous studies [30–33], we also tested our models on the relatively unbiased LIT-PCBA dataset reported by Tran-Nguyen et al. [31, 32] LIT-PCBA was specially designed for VS and ML benchmark. It was unbiased by the asymmetric validation embedding (AVE) procedure, and contains 15 diverse targets with a total of 7844 active and 407,381 inactive compounds. Five targets, including ADRB2, ESR1_ago, OPRK1, IDH1, and PPARG, were removed in this study because the number of the active compounds for each target is less than 50, which may not provide sufficient information for a ML algorithm. Given that multiple protein structures are available in LIT-PCBA, the ligands were docked into all the available crystal structures of each target, and the P-values between docking scores of actives and decoys were subsequently calculated. The crystal structure with the lowest P-value for each target was retained for further descriptor generation. The statistical significance data can be found in Additional file 1: Figure S1, and the details of the two datasets used in this study are shown in Table 1. Besides, another dataset (Dataset III) was constructed by expanding the decoy sets of 7 targets in LIT-PCBA (i.e., ALDH1, FEN1, GBA, KAT2A, MAPK1, PKM2, and VDR) using compounds randomly extracted from the ChemDiv database.

**Table 1:** Information of the diverse subset of DUD-E and LIT-PCBA after preparation

Target	PDB_ID	Active	Decoys	Total	Active/decoys %
akt1	3cqw	674	33,484	34,158	2.01
ampc	1l2s	95	5831	5926	1.63
cp3a4	3nxu	840	19,918	20,758	4.22
cxcr4	3odu	871	8779	9650	9.92
gcr	3bqd	179	8649	8828	2.07
hivpr	1xl2	3476	58,731	62,207	5.92
hivrt	3lan	867	23,265	24,132	3.73
kif11	3cjo	334	13,313	13,647	2.51
ALDH1	5l2n	7554	149,358	156,912	5.06
ESR1_ant	2iog	111	6189	6300	1.79
FEN1	5fv7	696	502,274	502,970	0.14
GBA	3ril	319	423,463	423,782	0.08
KAT2A	5h86	306	494,569	494,875	0.06
MAPK1	4qte	442	91,185	91,627	0.49
MTORC1	4dri	157	42,223	42,380	0.37
PKM2	3me3	665	301,123	301,788	0.22
TP53	3zme	113	5779	5892	1.96
VDR	3a2i	678	216,464	217,142	0.31

### Preparation and ligand docking

All the structures in the Datasets I and II were first prepared using the *Protein Preparation Wizard* module in *Schrödinger* [34] More specifically, the bond orders were assigned and the hydrogen was re-added. Then, each protein structure was repaired by creating necessary bonds to proximal sulfurs, filling missing side chains and missing loops, and optimizing the hydrogen bonding (H-bond) network with the OPLS3 force field [35] *PROPKA* was used to generate the protonation states of residues at pH = 7.0 and *Epik* was employed to generate the ionized states of heteroatoms [36, 37] The small molecules in the benchmark datasets were prepared using the *LigPrep* module, including correcting the hydrogen atoms, and generating the protonation states at pH = 7.0 ± 2.0, stereoisomers and tautomers enumeration [34].

The binding site for each target was determined based on the position of the co-crystallized ligand [2, 38] Then, all the prepared ligands were docked into the corresponding structures by *Glide* SP docking or *Smina* docking, and only the top-1 scored binding pose was retained for each ligand. Although the top-1 scored binding pose may not be the correct binding conformation, retaining the top-1 scored binding pose for modelling has the following advantages: (1) it significantly enhances the computational efficiency, which is crucial when screening large ligand libraries [14, 39–41], (2) it aligns with common practice in the field as evidenced by previous research [23, 24, 42, 43], and (3) it may improve the generalization ability of the machine learning model by training on docked poses rather than crystalized structures, thereby potentially improving its performance.

### Scoring functions and energy terms

In this study, 15 traditional scoring functions were used to re-score the binding poses obtained from docking, and the descriptors were extracted from the output scoring files [2–4, 38, 42, 44–49] The detailed information of the SFs was listed in Table 2 and Additional file 1: Part1. According to the physical principles, the energy terms from the 15 SFs can be roughly categorized into the following groups:

**Table 2:** Information of the scoring functions and the corresponding energy terms

Scoring Functions	Energy terms	Number of descriptors
Autodock	${E}_{vdW}$, ${E}_{\mathrm{h}bond}$, ${E}_{elec}$, ${E}_{tor}$,${E}_{sol}$	5
Affiscore	${E}_{polar}$, ${E}_{sol}$,${E}_{\mathrm{h}ydrophobic}, {E}_{entropy}$	4
Asp	${E}_{internal}$, ${E}_{clash}$,${E}_{map}$	3
Chemscore	${E}_{\mathrm{h}bond}$, ${E}_{entropy}$, ${E}_{internal}$, ${E}_{clash}$, ${E}_{\mathrm{h}ydrophobic}$,${E}_{Metal}$	6
DSX	${E}_{pair}$, ${E}_{internal}$,${E}_{sol}$	3
GalaxyDock BP2 score	${E}_{vdW}$, ${E}_{\mathrm{h}bond}$, ${E}_{elec}$, ${E}_{sol}$, ${E}_{internal}$, ${E}_{\mathrm{h}ydrophobic}$,${E}_{drugscire}$	7
Goldscore	$\Delta {G}_{vdW}$, $\Delta {G}_{\mathrm{h}bond}$,$\Delta {G}_{internal}$	3
NNScore	${E}_{vdW}$, ${E}_{\mathrm{h}bond}$, ${E}_{\mathrm{h}ydrophobic}$, ${E}_{nn\_pair}$,${E}_{nn\_elec}$	5
ChemPLP	${E}_{\mathrm{h}bond}$, ${E}_{Metal}$, ${E}_{clash}$, ${E}_{plp}$,${E}_{internal}$	5
Smina	${E}_{vdW}$, ${E}_{\mathrm{h}bond}$, ${E}_{elec}$, ${E}_{\mathrm{h}ydrophobic}$, ${E}_{non\_hydrophobic}$,${E}_{sol}$	6
SMoG2016	${E}_{vdW}$, ${E}_{entropy}$,${E}_{KBP2016}$	3
Glide SP	${E}_{vdW}$, ${E}_{\mathrm{h}bond}$, ${E}_{elec}$, ${E}_{\mathrm{h}ydrophobic}$, ${E}_{Metal}$,${E}_{entropy}$	6
Glide XP	${E}_{vdW}$, ${E}_{\mathrm{h}bond}$, ${E}_{elec}$, ${E}_{\mathrm{h}ydrophobic}$, ${E}_{Metal}$, ${E}_{entropy}$,${E}_{\pi -cation}$	7
Vina	${E}_{vdW}$, ${E}_{entropy}$, ${E}_{\mathrm{h}bond}$,${E}_{\mathrm{h}ydrophobic}$	4
X-score	${E}_{vdW}$, ${E}_{entropy}$, ${E}_{\mathrm{h}bond}$,${E}_{\mathrm{h}ydrophobic}$	4
Total	${E}_{vdW}$, ${E}_{\mathrm{h}bond}$, ${E}_{elec}$, ${E}_{\mathrm{h}ydrophobic}$, ${E}_{sol}$, ${E}_{entropy}$, ${E}_{Knowledge}$, ${E}_{clash}$, ${E}_{Metal}$, ${E}_{internal}$, ${E}_{non\_hydrophobic}$,${E}_{nn\_pair}$	12

#### Van der Waals interaction

The van der Waals interaction in SFs usually refers to the non-bonded interactions that are not electrostatic, which is of great significance for prediction of protein–ligand binding [2, 38, 42, 46–53] In most SFs, it is described as the Lennard–Jones potential [38, 46, 53, 54]. However, empirical potential functions are also used to improve efficiency [2, 51].

#### Electrostatic interaction

Electrostatic energy gives a description of the potential of attractions or repulsions between polar atoms, and is calculated by the Coulomb potential function based on the partial atomic charges 2, 38, 42, 46, 51, 53].

#### Hydrogen bond interaction

Hydrogen bonding happens when a hydrogen atom covalently bound to an electronegative ‘‘donor’’ atom undergoes dipole–dipole attraction to an electronegative ‘‘acceptor’’ atom. It is generally denoted as D-H…A, with proper distance and angle for a donor–acceptor pair. As one of the most important interactions in biomolecular systems, H-bond interactions are explicitly included in most SFs [2, 4, 38, 42, 46, 48, 49, 51–53, 55].

#### Hydrophobic energy term

During the formation of a protein–ligand complex, the water molecules are released from the nonpolar molecular surface to the solvent, which is related to the hydrophobic effects. Multiple strategies have been introduced to calculate the hydrophobic term by considering the potential buried hydrophobic surface of the ligand, the number of the hydrophobic atom pairs, and whether a hydrophobic ligand atom is in a hydrophobic binding site [2, 4, 38, 42, 46, 48, 49, 51, 52].

#### Solvation effect

The solvation term calculates the free energy cost of breaking the interactions between solvent and protein/ligand upon ligand binding. Due to the difficulty of calculating this term, SFs usually simplify this term based on some hypotheses. For example, ligand binding to the pocket causes the variation in the number of H-bonds formed among protein, ligand and solvent, so one way to describe the solvation effect is to determine the difference of H-bond numbers before and after docking [38, 46, 53].

#### Entropy effect

During the docking process, the conformation of ligand and protein will be constrained by fixing their rotatable bonds. The entropy term, also called deformation effect, is associated with the change of flexibility of the protein or ligand in the binding process. However, in most cases, only ligand entropy is considered since the protein is usually treated as rigid in a docking system [2, 4, 47–49, 51, 52].

#### Clash effect

This term penalizes the irrational close contact between ligand and protein to prevent the generation of inappropriate geometries in docking [4, 55, 56].

#### Metal interaction

The metal-binding term is typically computed as a sum over all possible metal-ion acceptor pairs, where the acceptor is an atom in the ligand that is capable of binding to a metal [2, 51, 55].

#### Internal potential

This component usually refers to a physics-based energy term counting for the torsion energy of the ligand [4, 55, 56].

All the interactions mentioned above are the most frequently implemented terms in SFs. The other energy terms that only appear in specific SFs were summarized and provided in the Additional file 1.

### Model training

Three ML algorithms, namely SVM, RF and XGBoost, were applied to model MLSFs based on different feature combinations. During the training process, SVM was used to find the best hyperplane to divide the positive and negative samples with the help of kernel function, while RF and XGBoost were utilized to build a set of base estimators (decision trees) to make predictions. In order to obtain better results, a voting estimator that uses the majority vote or the average of the probabilities from the base estimators was then used. Hyper-parameters are parameters that may significantly influence the performance of models but cannot be learned through training. Therefore, *hyperopt*, a python package for hyper-parameter tuning, was used to find proper hyper-parameters to train better models. For SVM with the Radial Basis Function (RBF) kernel, the regularization parameter C (from 0.1 to 10, uniform distribution) and the kernel coefficient gamma (from 0.001 to 1, uniform distribution) were then optimized. For RF, the number of base estimators (from 100 to 300, interval = 10), the maximum depth of the tree (from 6 to 100, interval = 1), the number of features used to find the best split (‘sqrt’ or ‘log2’) and the minimum number of samples required at a leaf node (from 3 to 10, interval = 1) were optimized. Similarly, the number of base estimators and the maximum depth of the tree were tuned for XGBoost within the same range for RF. Besides, two different hyper-parameters in XGBoost, i.e., the step size shrinkage used in update (from 0.1 to 0.5, uniform distribution) and the L2 regularization parameter (from 0.5 to 3, uniform distribution), were also optimized.

In the training process, the datasets were first shuffled and split into the training set and test set at the ratio of 4:1 by stratified sampling according to the labels. The raw features were normalized to the Gaussian distribution with zero mean and unit variance, and the features with low variance were subsequently removed. Then a one-hundred-step hyper-parameter tuning was performed using *hyperopt*, and the tenfold cross validation (CV) was employed to evaluate the performance of models with different hyper-parameters. The model with the best hyper-parameter was regarded as the final model and assessed on the test set under different evaluation metrics.

### Evaluation metrics

Our models were designed to distinguish binders from nonbinders for given targets. In this study, three metrics, including the F1 Score, the area under the curve (AUC) of receiver operation characteristic (ROC) curve and the enrichment factor at the 1% level (EF_1%), were adopted to assess the screening power of MLSFs. The F1 Score (ranging from 1 to 0), which can be interpreted as a weighted average of precision and recall, is a balanced metric for classification. The ROC curve is plotted based on the sensitive and specificity under various thresholds, of which the AUC reflects the overall performance of classifiers in VS. The AUC value closer to 1 indicates the better overall predictive performance of the model, while a AUC value of 0.5 indicates a random prediction. The EF value is a widely used metric for validating the quality of VS protocol, which is defined as the proportion of active compounds identified by employing a certain VS strategy. The above-mentioned metrics are computed according to the formula, where TP represents true positive, FP represents false positive, TN represents true negative, and FN represents false negative.

1 $$\mathrm{precision}=\frac{TP}{TP+FP}$$

2 $$\mathrm{recall}=\frac{TP}{TP+FN}$$

3 $$\mathrm{F}1=\frac{2*(precision*recall)}{precision+recall}$$

4 $${\mathrm{EF}}_{1\%}=\frac{The\, number\, of\, actives\, at\, 1\%\, level/the\, number\, of\, molecules\, at\, 1\% level}{The \,total \,number \,of \,actives/the \,total \,number\, of\, molecules}$$

## Results and discussion

**Fig. 1:**
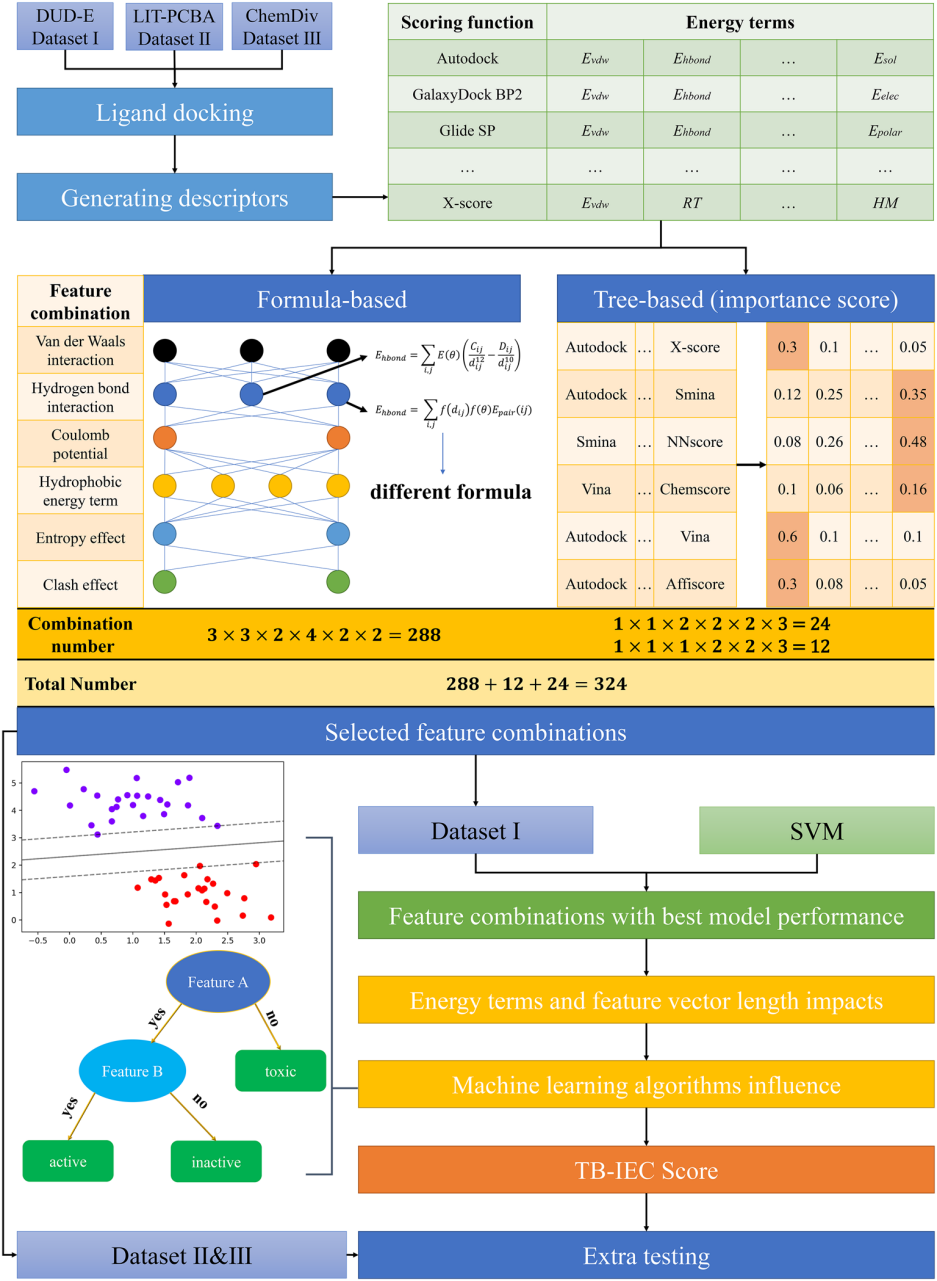
The workflow of this study, including dataset collection, ligand docking, descriptors generation, feature combination and experiments about best feature combination selection, impacts of energy terms, feature length and machine learning algorithms on model performance, the performance of TB-IECS on benchmarks and virtual screening

The complete workflow of this study is shown in Fig. 1. First, three datasets (i.e., Dataset I, Dataset II and Dataset III) were collected, and the protein–ligand complex structures were predicted by docking. The top-1 ranked binding complex for each ligand in Dataset I and Dataset II was selected for the generation of descriptors and rescored by 15 classical SFs (Table 2). The decomposed energy terms from 15 SFs were then used as the descriptors for the construction of MLSFs. Before MLSFs modelling, the energy terms were grouped in terms of their formula and physical principles. Two distinct feature combination strategies were devised to formulate theory-driven feature combinations, aiming to encompass as many interaction types as possible, while avoiding feature redundancy. The first strategy amalgamates features of varying interaction types across different formulas, yielding 288 combinations (Table 3). The alternative approach provides 36 feature combinations, adhering to the importance scores generated by a tree-based feature selection protocol (Table 3). Thus, a cumulative 324 feature combinations were obtained for further analysis. Next, the SVM algorithm was utilized for training and testing based on the 324 feature groups of Dataset I. According to the performances of the 324 models, the 5 best feature combinations were selected for further investigation of the influence of feature vector length, physiochemical energy component, and ML algorithms on the model performance. Finally, we proposed a new TB-IECS developed based on the energy terms obtained from Smina, NNScore 2 via XGBoost algorithm, and the screening power of TB-IECS was further assessed on Dataset I, Dataset II and Dataset III.

**Table 3:** Combination of energy terms

Combination	VdW	Hbond	Elec	Hydrophobic	Entropy	Clash	Combination number
Formula	GalaxyDo-ck BP2 Goldscore Smina	GalaxyDo-ck BP2 Goldscore Smina	GalaxyDo-ck BP2 NNScore	Chemscor-e Affiscore X-score Glide XP	SMoG20-16 Glide XP	Chemscor-e ChemPL-P	288
Tree-sum	Glide SP	ChemPL-P	Autodock Glide XP	Glide XP Affiscore	Chemscor-e SMoG2016	Chemscor-e ChemPL-P ASP	24
Tree-mean	Smina	Smina	NNScore	Glide XP Smina	SMoG20-16 Glide XP	Chemscor-e ChemPL-P ASP	12

VdW, Hbond, and Elec represent the Van der Waals interaction, the Hydrogen bond interaction, and the Coulomb potential, respectively

### The performance of models trained on different features

The energy terms decomposed from all the 15 SFs provide comprehensive information of protein–ligand interactions. Three approaches (i.e., Formula-based, Tree-based-sum, and Tree-based-mean) were utilized to generate 324 designed combinations of energy terms (Table 3) that were presumed to give more accurate description of protein–ligand interactions. Formula-based method aim to combinate features in terms of formula representation and physicochemical properties. The energy terms decomposed from the 15 SFs were first categorized into 12 groups according to their physicochemical representation, namely van der Waals interaction, electrostatic interaction, H-bond interaction, hydrophobic effect, entropy, clash effect, solvation effect, metal interaction, internal potential, polar interaction, non-hydrophobic term, and knowledge-based term, where each group contained at least one formula expression. To avoid combinatorial explosion, only six frequently used energy terms (van der Waals interaction, electrostatic interaction, H-bond interaction, hydrophobic effect, entropy and clash effect) were involved in the feature combination, while the others were retained in default. For each of the six terms, the corresponding terms from various SFs with different formula were selected, resulting in 288 formula-based combinations of energy terms.

**Fig. 2:**
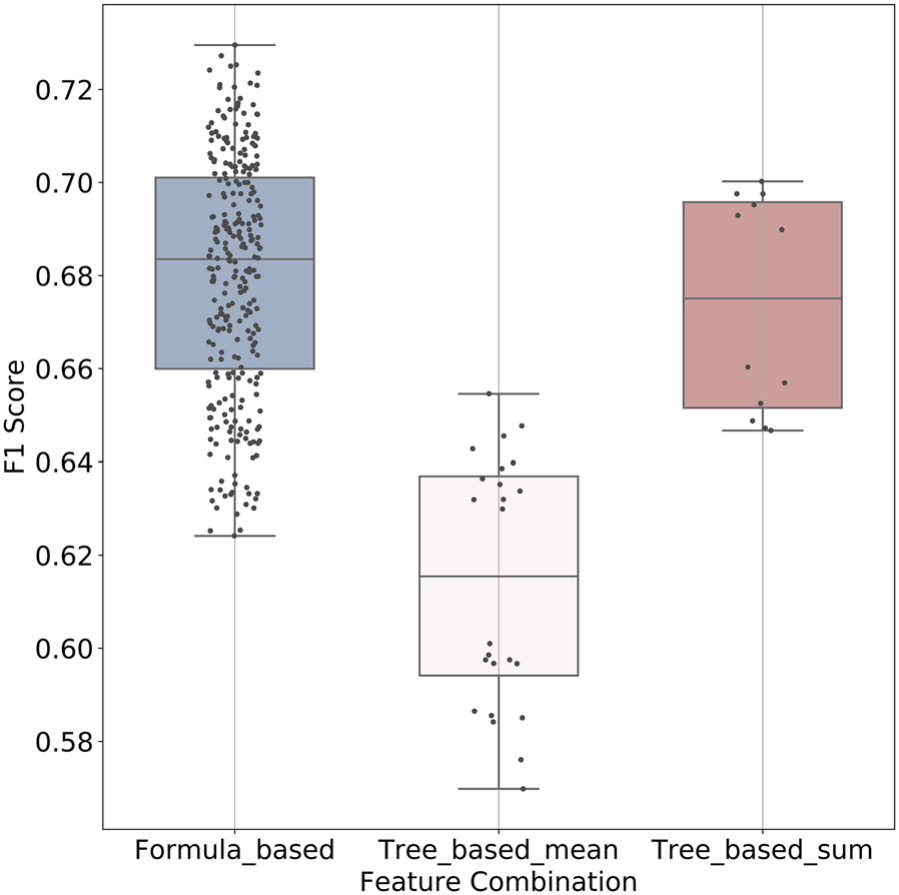
The performance of the 324 models. The performance of each model is listed in the (Additional file 1: Tables S1, S2, S3, S4)

Tree-based feature selection is one of the most frequently-used technologies in feature engineering, and it predicts and selects important features using the RF algorithm. Hence, both the Tree-based-sum approach and Tree-based-mean approach were used to evaluate the significance of each SF for specific interactions. Tree-based-sum means that the importance of the energy terms was summed, while the Tree-based-mean approach calculates the mean value of the importance of the energy terms. When multiple energy terms from distinct SFs describe the same interaction, and yield similar importance scores in different formulas representation, all such terms will be retained for feature combination. *Glide* and *Vina* docking were used for the generation of the binding poses, and the feature importance of the SFs in terms of various energy terms was calculated and summarized in the Supplementary Material (Additional file 1: Figure S2). The predicted feature importance varied when using different docking programs, which was mainly influenced by the distinct binding poses of the ligand from docking. Besides, different SFs also brought significant fluctuations on the prediction of feature importance. For example, *ChemPLP* rather than *GalaxyDock BP2* achieved the highest importance score of the H-bond term (Additional file 1: Figure S2B) when docking poses were generated by *Glide*. Given that, 24 and 12 feature combinations were generated by Tree-based-mean and Tree-based-sum, respectively. In total, 324 groups of features were used to construct MLSFs based on SVM, and the performances of the models were shown in Fig. 2. The models were all trained using the theory-based features, but the predictive performance differed among feature combinations, suggesting that a certain combination of interactions is of great significance to model accuracy and efficiency. According to the results, the Formula-based models generally achieved better performance (average F1 Score = 0.680) compared to the Tree-based methods.

### The performance of models trained on theory-based features and on single SFs

**Fig. 3:**
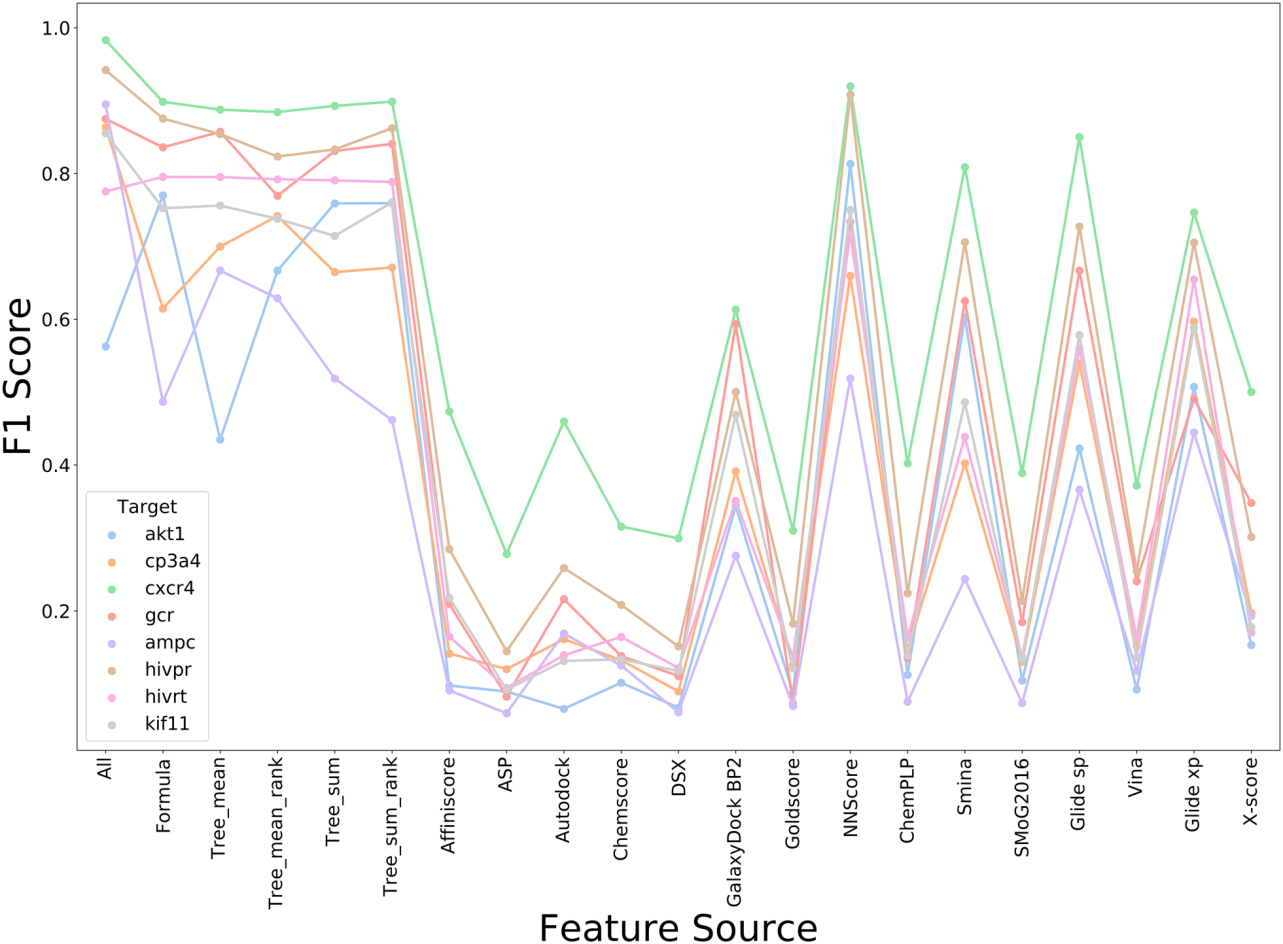
The performance of models trained on theory-based features and on singe SFs and tested on Dataset I.(1) All: the model trained on all the energy components of the 15 SFs with feature selection, (2) Formula: the best model among the models using Formula-based feature combinations, (3) Tree-mean and Tree-sum: the best model among the models using Tree-based feature combinations, (4) Tree-*-rank: models trained on the features ranked first according to the importance, (5) others: models built on single SFs’ energy components

The best feature combination from each group (i.e., Formula-based, Tree-based-sum, and Tree-based-mean) was retained for further assessment (Table 4). Besides, according to the predictions of feature importance from Tree-based methods, the energy components with the highest importance scores were combined, producing 2 other feature combinations (i.e., Tree-based-mean-rank and Tree-based-sum-rank). Tree-based-mean/sum and the Tree-based-mean/sum-rank combinations share the same SFs for four out of six energy terms (the van der Waals, electrostatic, H-bond and hydrophobic effect terms). We also constructed models trained on all features (all the energy components from the 15 SFs), and further compared with the models trained on the selected five theory-based feature combinations. The F1 Scores of all the models were shown in Fig. 3. The all-features model achieved the best performance for almost all the tested targets. Our theory-based feature-combination models also showed satisfactory accuracy, and there were no significant differences in F1 Scores among models for most targets, especially the models using the Formula-based and Tree-based-sum (rank) feature combinations. In contrast, the differences in F1 Scores between the theory-based models and most single-SF models (Fig. 3) reached statistical significance, suggesting that the theory-based feature combination generally improved model performance. It was not surprising since the theory-based features contained a more comprehensive description of interactions between the protein and ligand than the energy components from a single SF. Of note, several single SFs (*NNScore*, *Smina*, and Glide SP/XP) with relatively larger numbers of descriptors also achieved competitive performance, indicating that the feature vector length could also affect model performance.

**Table 4:** Theory-based feature combination with the best model performance

Combination	VdW	Hbond	Elec	Hydrophobic	Entropy	Clash
Formula	GalaxyDoc-k BP2	Goldscor-e	NNScore	X-score	SMoG2016	Chemscore
Tree-mean	Glide SP	ChemPL-P	Autodoc-k	Glide XP	SMoG2016	ASP
Tree-sum	Smina	Smina	NNScore	Glide XP	Glide XP	ChemPLP
Tree-mean-rank	Glide SP	ChemPL-P	Autodoc-k	Glide XP	Chemscore	Chemscore
Tree-sum-rank	Smina	Smina	NNScore	Glide XP	SMoG2016	Chemscore

VdW, Hbond, and Elec represent the Van der Waals interaction, the Hydrogen bond interaction, and the Coulomb potential, respectively

### The impact of interaction types and feature vector length on model performance

**Fig. 4:**
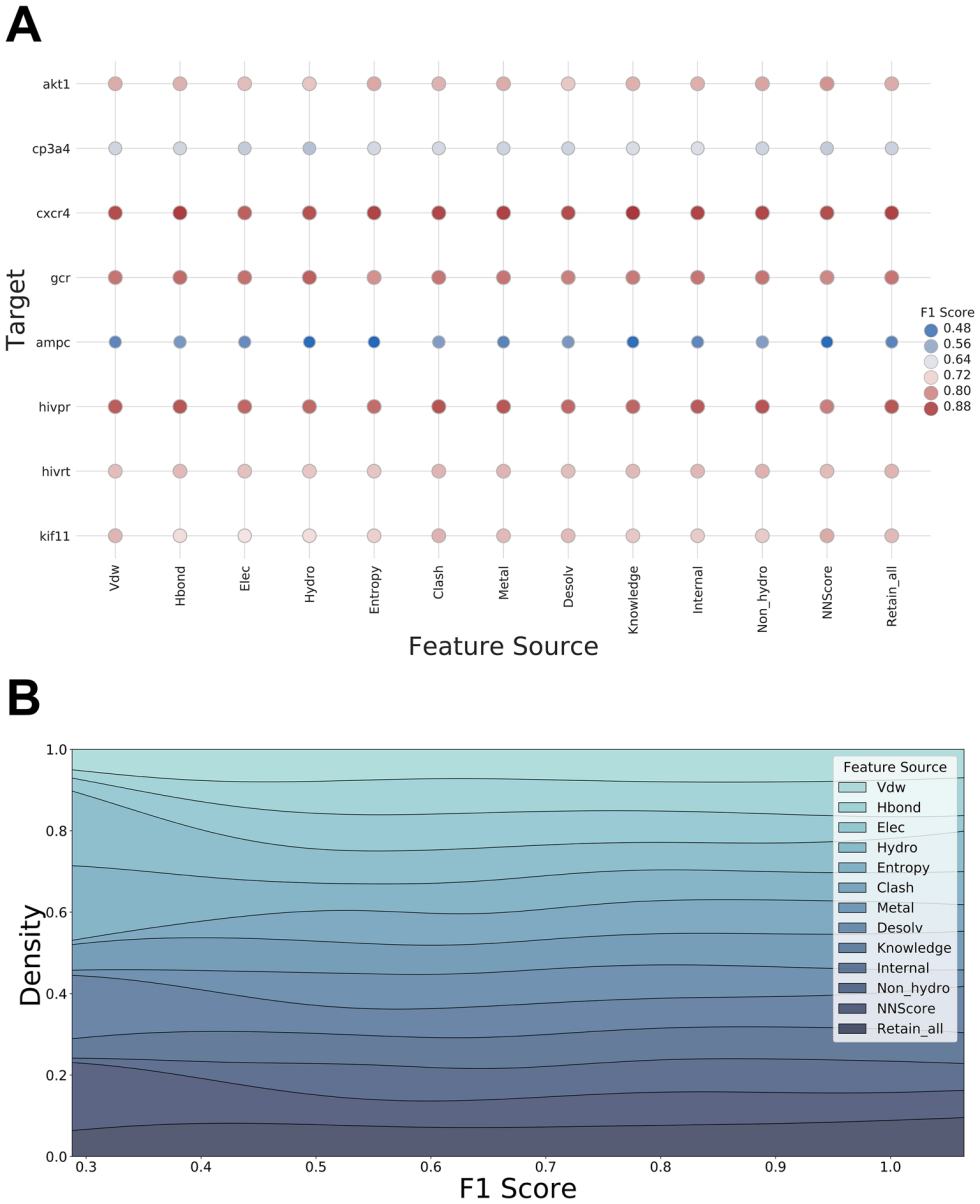
The impact of interaction energy terms on model performance: the performance of models trained on features lack of specific interactions. For example, the column ‘Vdw’ represents the performance of the model trained on the features without a description of Van der Waals’s interaction. **A**: heatmap of the performance of models with different features. **B**: the distribution of the performance of models with different features

In order to study the influence of different interaction terms on model performance, a series of feature combinations, where one type of interaction terms was removed from each Formula-based feature combination, were subsequently created. As shown in Fig. 4A, no significant decrease in F1 Score was observed when any one type of the interaction term was missing, and the distributions of F1 scores kept unchanged (Fig. 4B). The models constructed based on the hybrid theory-based feature combinations showed superiority over most SFs, since they contained a considerable number of descriptors that made them insensitive to the loss of a single type of interaction terms. For example, the loss of H-bond term may be compensated by the augmented description of electrostatic interaction. Another example is the hydrophobic effect, which is well-acknowledged as an entropy-driven process, associated with both the hydrophobic effect term and the entropy term. The loss of either term may have limited impact on overall efficiency.

**Fig. 5:**
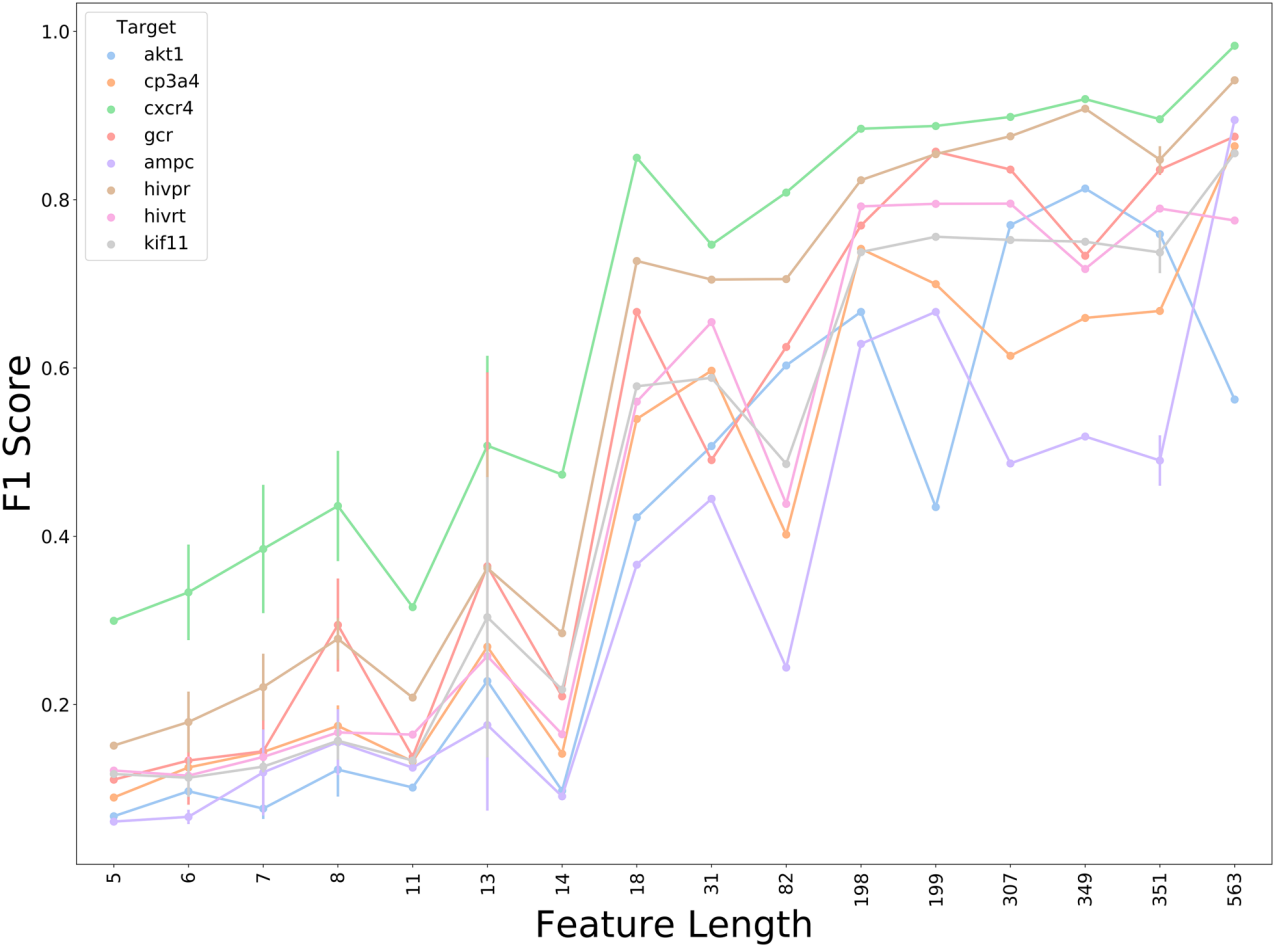
The relationship between feature vector length and model performance. The performance of the model trained on features with various lengths on Dataset I. Some feature combinations with the same feature vector length were merged in the figure

**Fig. 6:**
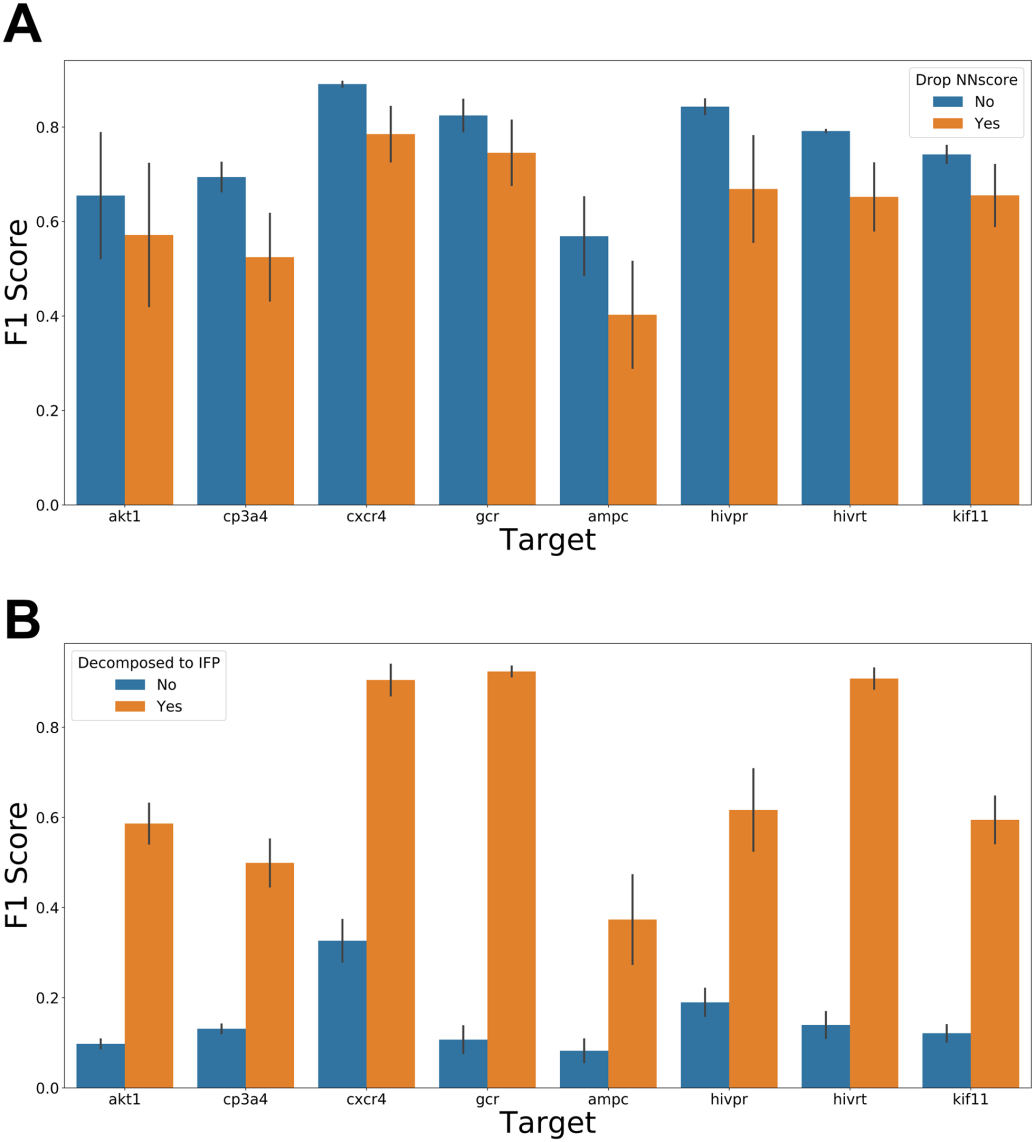
The impact of feature vector length and interaction energy terms on model performance. **A**: the change of the model performance after feature vector length reduction, **B**: the change of the model performance after feature vector length increasing. The feature reduction was implemented by remove descriptors of NNscore from raw feature combinations while the feature vector length was increased through decomposed the raw energy terms into interaction fingerprints

We next evaluated the impact of the feature vector length on model performance. As shown in Fig. 5**,** the F1 Scores increased in all the tested targets as the number of features increased. A final steady state was reached after the number of features increased to around 200, where the use of more descriptors failed to improve accuracy. For further confirmation of the relation between model performance and descriptor length, we removed the *NNScore* descriptors from the theory-based features (i.e., Tree-mean, Tree-sum, Tree-mean-rank and Tree-sum-rank) to decrease the feature number, and decomposed the energy terms from the SFs in *GOLD* (i.e., Asp, Chemscore, Goldscore and ChemPLP) into the interaction fingerprints (IFP) to increase the feature number. The performances of the models built on these features were shown in Fig. 6 and Additional file 1: Figure S3, where the performances of the models turned down as the feature vector length decreased. On the one hand, the model performance was enhanced after the energy components were decomposed into IFPs, indicating that the model performance could be improved by transforming coarse-grained descriptors into fine-grained descriptors that captured more detailed but distinct information of protein–ligand interactions at atomic and residual levels, and avoided the issue of descriptor redundancy. On the other hand, the use of NNScore with complementary descriptors was found beneficial to improve model performance.

### The impact of machine learning algorithms on model performance

**Fig. 7:**
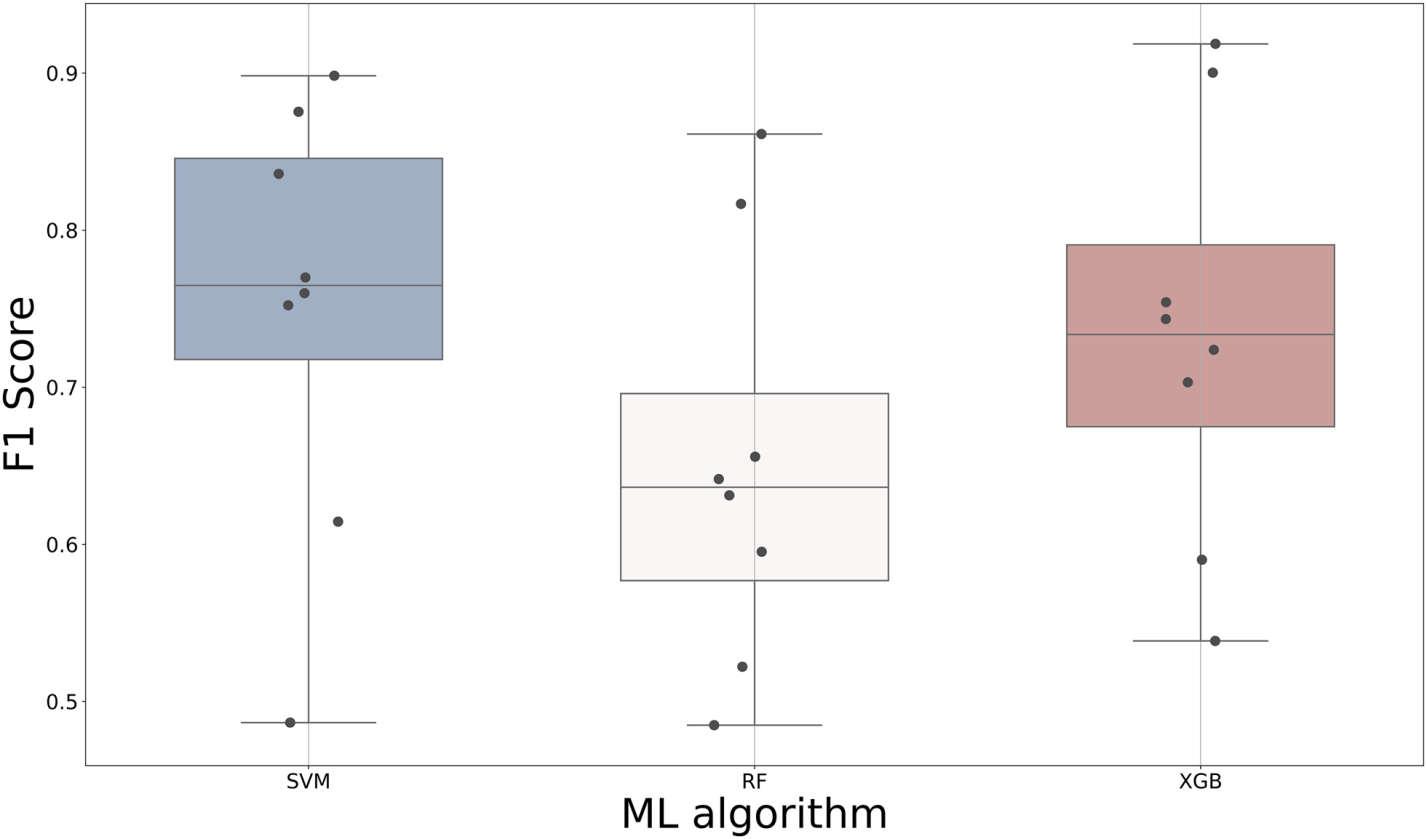
The impact of machine learning algorithms on model performance

To explore the influence of ML algorithms on model performance, SVM, RF and XGBoost were used for modeling based on formula-based features. According to the results shown in Fig. 7, the SVM models were the best based on the average F1 Score while the RF models showed the worst performance. However, there was no significant difference among the models using the same ML algorithm. Considering that the XGBoost models showed competitive performance with the SVM models but calculated faster than the SVM models, the XGBoost algorithm was used for further modeling.

### The performance of models trained on better descriptors and ML algorithms

Based on the above results, we selected long theory-based features and XGBoost algorithms and tried to construct an accurate MLSF. As shown in Fig. 6B, decomposing the single energy terms into the contribution scores (IFPs) of protein residues and ligand atoms could dramatically improve the model performance. The *ChemPLP ifp* was used as a new theory-based feature combination because the model built on this feature performed best among the 8 models in Additional file 1: Figure S3B. In addition, the energy components of *NNScore* and *Smina* were also implemented in the new theory-based features because these two SFs provided a number of useful complementary descriptors. The interaction components (i.e., entropy term and knowledge-based potential) that were not provided by *ChemPLP ifp*, *NNScore* and *Smina* were supplemented by *SMoG2016* [47] As *ChemPLP* is one of the SFs in the commercial software *GOLD*, the model may be restricted for academic use, and the speed of generating *ChemPLP ifp* is very slow. For this reason, we introduced feature combinations consisting of descriptors only from *NNScore* and *Smina*, which not only provided a full description of the protein–ligand interaction but also showed faster calculating speed (shown in Table 5).

**Table 5:** New theory-based feature combination

Combination	VdW	Hbond	Elec	Hydrophobic	Entropy	Clash
Chemplp_smina_nn_smo	Smina	ChemPLP	NNScore	Smina	SMoG2016	ChemPLP
Smina_nn	Smina, NNScore	Smina, NNScore	Smina, NNScore	Smina, NNScore	Smina	–

VdW, Hbond, and Elec represent the Van der Waals interaction, the hydrogen bond interaction, and the Coulomb potential, respectively

The XGBoost algorithm was further utilized to construct the target-specific SF and the model was then tested on the DUD-E set (Table 6). In general, the models trained on the new theory-based feature combinations outperformed the classical SFs. The *Smina_nn* model performed the best under most circumstances, and the *Chemplp_smina_nn_smo* model also achieved competitive performance. Therefore, *Smina_nn* model was retained as our final model named as the theory-based interaction energy component (TB-IEC) Score.

**Table 6:** Model performance on DUDE

Targets	Chemplp_smina_nn_smo			Smina_nn (TB-IEC Score)			Glide_SP		DUDE_Dock	
	ROAUC	F1_Score	EF_1%	ROAUC	F1_Score	EF_1%	ROAUC	EF_1%	ROAUC	EF_1%
akt1	0.976	0.733	**50.896**	0.993	**0.833**	50.610	0.584	5.054	0.720	29.4
ampc	0.892	0.462	36.228	0.979	**0.519**	**41.610**	0.558	1.057	0.789	8.3
cp3a4	0.941	0.618	24.970	0.993	**0.789**	**25.000**	0.597	2.985	0.631	2.4
cxcr4	0.994	0.927	11.063	1.000	**0.986**	11.090	0.639	2.885	0.902	**17.5**
gcr	0.997	0.727	**50.286**	0.995	**0.742**	49.060	0.746	18.074	0.439	8.9
hivpr	0.995	0.903	17.894	0.999	**0.970**	**17.900**	0.531	2.359	0.596	4.7
hivrt	0.944	0.662	27.855	0.959	**0.746**	**27.900**	0.563	7.125	0.644	6.5
kif11	0.955	0.660	**41.273**	0.973	**0.825**	40.750	0.635	16.524	0.769	34.5

The best performance under the perspective of three metrics for each target are shown in bold

### The performance of TB-IECS on LIT-PCBA and virtual screening

**Fig. 8:**
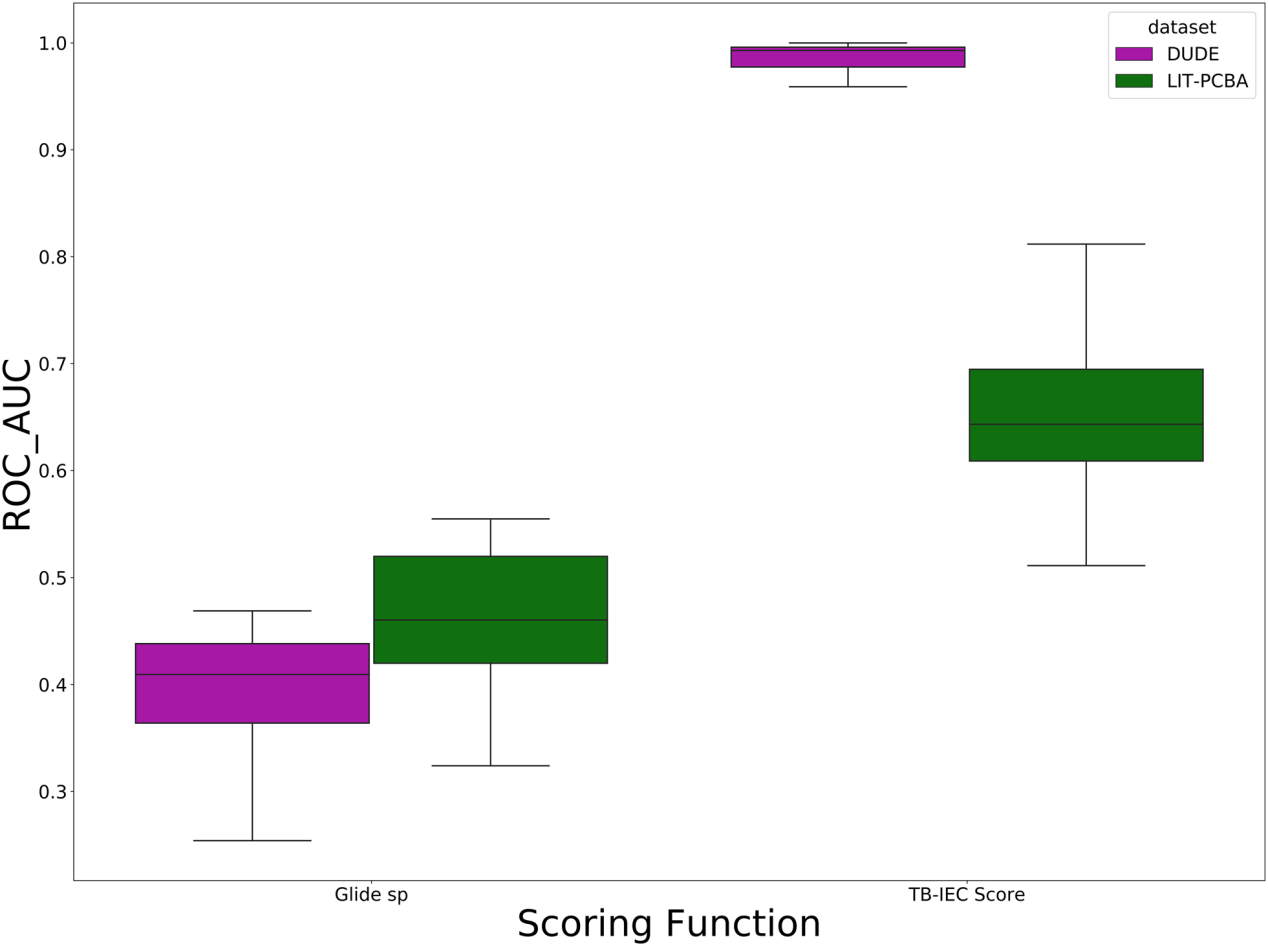
The performance of TB-IECS and Glide SP on DUD-E (Dataset I) and LIT-PCBA (Dataset II)

**Fig. 9:**
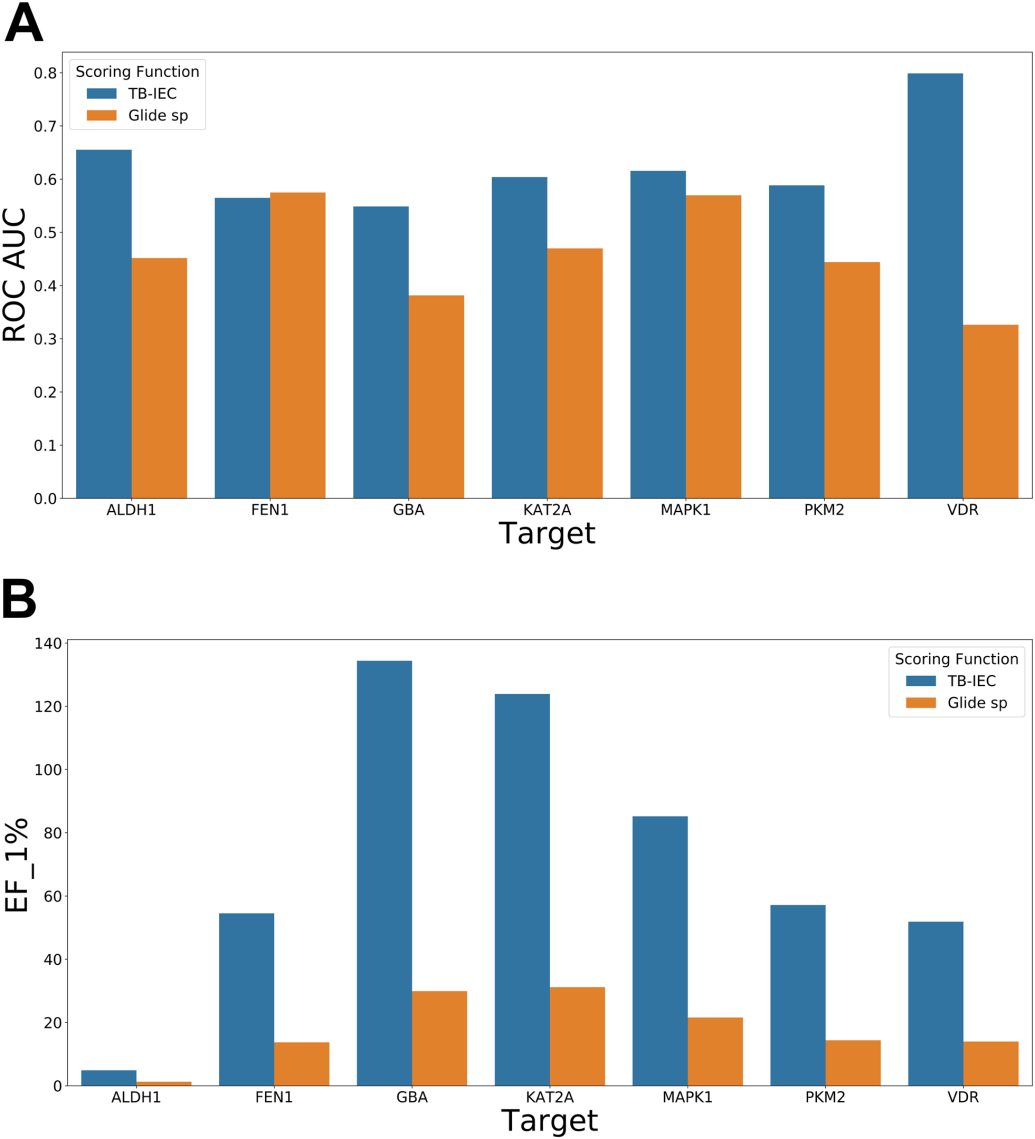
The performance of TB-IECS on Dataset III. **A**: the ROC_AUC of TB-IECS and Glide SP, **B**: the EF of TB-IECS and Glide SP

Although TB-IECS showed outstanding performance on DUD-E set (Dataset I), further validations on other datasets were still needed due to the existing hidden bias in the DUD-E set that had been described previously [31, 32] LIT-PCBA (Dataset II), introduced by Tran-Nguyen et al. in 2020, is an unbiased dataset for MLSF assessment consisting of 15 diverse targets with a total of confirmed 7844 active and 407,381 inactive compounds. As shown in Fig. 8, the AUC value of TB-IECS dropped from 0.986 (on DUD-E set) to 0.652 (on LIT-PCBA set), indicating a sharp decrease in accuracy, but was still significantly higher than that of Glide SP. Of note, the numbers of actives and decoys in LIT-PCBA set were extremely unbalanced, which might have a huge impact on model accuracy. Besides, TB-IECS exhibited superiority in early recognition compared to Glide SP that showed poor screening power with EF_(1,2,5%)_ equaling to 0 (Table 7). In order to further explore the screening power of TB-IECS, seven TB-IECS models were specifically trained for seven different targets and used to screen the ChemDiv database (Dataset III). More than 2 million ligands in the ChemDiv database were prepared and docked into corresponding proteins, followed by the TB-IECS screening. As shown in Fig. 9, the performance of TB-IECS on Dataset III was similar to that on LIT-PCBA in terms of AUC value. As for EF_(1%)_, both TB-IECS and Glide SP showed improved performance on Dataset III than on LIT-PCBA, but TB-IECS was also more effective than Glide SP. In brief, TB-IECS exhibited potential ability in virtual screening and outperformed Glide SP in different evaluations.

**Table 7:** Model performance on LIT-PCBA

Targets	TB-IEC score				Glide_SP	
	ROAUC	F1_Score	EF_1%		ROAUC	EF_1%
ALDH1	0.610	0.031	8.960		0.482	0
ESR1_ant	0.697	0	2.80		0.324	0
FEN1	0.812	0	1.143		0.386	0
GBA	0.689	0	0		0.521	0
KAT2A	0.523	0	1.299		0.517	0
MAPK1	0.697	0	0		0.429	0
MTORC1	0.511	0	0		0.555	0
PKM2	0.632	0	1.198		0.439	0
TP53	0.654	0	7.279		0.417	0
VDR	0.609	0	0.543		0.530	0

## Conclusion

In this study, we collected multiple energy components from 15 classical SFs describing the important non-bonded interactions in protein–ligand complexes and regrouped these energy terms to form a theory-based feature combination. A target-specific MLSF named TB-IECS was further constructed with strong screening power. A total of 324 theory-based feature combinations were generated based on two methods (Formula-based and Tree-based) and were used for modeling. We found that selection of appropriate feature combinations based on formula representations and physicochemical meanings could improve model performance, and 5 theory-based feature combinations were subsequently identified and used for model construction. We further explored the impact of interaction type, feature vector length and ML algorithms on model performance, and finally proposed our TB-IECS.

During the modeling process, we found that the feature vector length had a great influence on model efficiency. The model performance improved as the number of features increased. A final steady state was reached after the number of features increased to around 200, where the use of more descriptors did not help improve accuracy. Removing any one interaction descriptor from the feature combination had little influence on model performance when using long feature vector length. Further use of the decomposed energy terms from SFs that provided detailed information of protein–ligand interactions dramatically improved the performance of MLSF. However, no significant difference of the overall performance was found among ML algorithms including SVM, RF, and XGBoost.

Recently, several high-performance MLSFs by introducing atom features from graph neural networks were constructed and exhibited signs of great promise.^22, 57^ In this work, TB-IECS utilized pairwise atom features that allowed for effective capture of the energetic patterns in protein–ligand recognition. Validations on DUD-E set, LIT-PCBA set and real-scenario VS suggested that TB-IECS showed much better performance than most conventional SFs, and significantly increased the low early recognition rates by classical SFs. Besides, TB-IECS performed far better than the MLSF constructed on energy terms from single SFs in terms of F1 Score, suggesting the superiority of using theory-based feature combination in MLSFs. Overall, TB-IECS represented as an accurate MLSF method and showed great promise in VS applications.

## Supplementary Information

**Additional file1: Table S1.** The name and scoring functions of Formula-based feature combination. **Table S2.** The name and scoring functions of Tree-based-mean feature combination. **Table S3.** The name and scoring functions of Tree-based-sum feature combination. **Table S4.** Performance of the models built on theory-based features. **Figure S1.** The frequency distribution plots of docking score of different pdb structures for targets **A** ALDH1, **B** ESR1_ant, **C** FEN1, **D** GBA, **E** KAT2A, **F** MAPK1, **G** MTORC1, **H** PKM2, **I** TP53 and **J** VDR. **Figure S2.** The importance of various scoring functions for different interactions **A** Van der Waals interaction, **B** Hydrogen bond interaction, **C** Coulomb potential, **D** Hydrophobic energy term, **E** Entropy effect, and **F** Clash effect. **Figure S3.** The impact of feature-length on model performance. **A**: the change of the model performance after feature-length reduction, **B**: the change of the model performance after feature-length increase.

## Data Availability

The datasets are available at http://dude.docking.org/ and http://drugdesign.unistra.fr/LIT-PCBA, respectively. The source code is available at https://github.com/schrojunzhang/TB-IEC-Score.
