# Supplementary material for: TB-IECS: an accurate machine learning-based scoring function for virtual screening
Source: J Cheminform. 2023 Jul 4;15:63. doi: 10.1186/s13321-023-00731-x (PMC10320911; doi:10.1186/s13321-023-00731-x)
Supplement: Supplementary file 1 — Additional file1: Table S1. The name and scoring functions of Formula-based feature combination. Table S2. The name and scoring functions of Tree-based-mean feature combination. Table S3. The name and scoring functions of Tree-based-sum feature combination. Table S4. Performance of the models built on theory-based features. Figure S1. The frequency distribution plots of docking score of different pdb structures for targets A ALDH1, B ESR1_ant, C FEN1, D GBA, E KAT2A, F MAPK1, G MTORC1, H PKM2, I TP53 and J VDR. Figure S2. The importance of various scoring functions for different interactions A Van der Waals interaction, B Hydrogen bond interaction, C Coulomb potential, D Hydrophobic energy term, E Entropy effect, and F Clash effect. Figure S3. The impact of feature-length on model performance. A: the change of the model performance after feature-length reduction, B: the change of the model performance after feature-length increase. [file 13321_2023_731_MOESM1_ESM.docx]

**Supporting Information**

**TB-IECS: An accurate and efficient machine learning-based scoring function for virtual screening**

Xujun Zhang, Chao Shen, Dejun Jiang, Jintu Zhang, Qing Ye, Lei Xu, Peichen Pan^*^, Yu Kang^*^

*Innovation Institute for Artificial Intelligence in Medicine of Zhejiang University, College of Pharmaceutical Sciences, Zhejiang University, Hangzhou 310058, Zhejiang, China*

*Institute of Bioinformatics and Medical Engineering, School of Electrical and Information Engineering, Jiangsu University of Technology, Changzhou 213001, China*

**Corresponding authors**

**Yu Kang**

E-mail: [yukang@zju.edu.cn](mailto:yukang@zju.edu.cn)

**Peichen Pan**

E-mail: [panpeichen@zju.edu.cn](mailto:panpeichen@zju.edu.cn)

**Part 1. Energy terms used in this study**

**1.1 Autodock[1-3]**

$$\left( 1 \right) SF=W_{1}E_{vdW}+W_{2}E_{hbond}+W_{3}E_{elec}+W_{4}N_{tor}+W_{5}E_{sol}$$

$$\left( 2 \right) E_{vdW}=\sum_{i,j} \left( \frac{A_{ij}}{r_{ij}^{12}}-\frac{B_{ij}}{r_{ij}^{6}} \right)$$

$$\left( 3 \right) E_{hbond}=\sum_{i,j} E\left( t \right)\left( \frac{C_{ij}}{r_{ij}^{12}}-\frac{D_{ij}}{r_{ij}^{10}} \right)$$

$$\left( 4 \right) E_{elec}=\sum_{i,j} \left( \frac{q_{i}q_{j}}{\varepsilon\left( r_{ij} \right)r_{ij}} \right)$$

$$\left( 5 \right) E_{sol}=\sum_{i,j} \left( S_{i}V_{j}+S_{j}V_{i} \right)e^{\left( -{r_{ij}^{2}}/{2\sigma^{2}} \right)}$$

where i, j is the index of ligand atom and protein atom, respectively; r_ij_ is the distance between atom i and atom j; A_ij_, B_ij_, C_ij_ and D_ij_ are parameters calculated from atom i and atom j; q_i_, q_j_ are partial atomic charges;$\varepsilon$ is the dielectric constant of bulk water at 25 ℃; $N_{tor}$ is the number of sp3 bonds in the ligand; (5) is a the desolvation term, evaluating the percentage of volume around the ligand atom that is occupied by protein atoms.

**1.2 Vina[4]**

$$\left( 1 \right) SF=\frac{C_{inter}}{1+wN_{rot}}$$

$$\left( 2 \right) C_{inter}=w_{1}{gauss}_{1}+w_{2}{gauss}_{2}+w_{3}repulsion+w_{4}hydrophobic+w_{5}hbonding$$

$$\left( 3 \right) {gauss}_{1}(d)=e^{-\left( d/{0.5Å} \right)^{2}}$$

$$\left( 4 \right) {gauss}_{2}\left( d \right)=e^{-\left( {(d-3Å)}/{2Å} \right)^{2}}$$

$$\left( 5 \right) repulsion=\left\{ \begin{aligned} d^{2}, if d<0 \\ 0, if d\geq0 \end{aligned} \right.$$

$$\left( 6 \right) hydrophobic=\left\{ \begin{aligned} 1, if d<0.5Å \\ 1.5-d, if 0.5Å\leq d\leq1.5Å \\ 0, if d>1.5Å \end{aligned} \right.$$

$$\left( 7 \right) hbonding= \left\{ \begin{aligned} 1, if d<-0.7Å \\ -\frac{10d}{7}, if-0.7Å\leq d\leq0Å \\ 0, if d>0Å \end{aligned} \right.$$

where $d$ is the distance between protein atom and ligand atom.

**1.3 Smina[5]**

$$\left( 1 \right)SF=W_{1-31}gauss+W_{32-39}repulsion+W_{40}vdW+W_{41-42}electrostatic+W_{43-45}hbond+W_{46-49}hydrophobic+W_{50}non\_hydrophobic+W_{51-52}solvation+W_{53-58}counts$$

$$\left( 2 \right) gauss\left( i, j, d \right)=e^{\left( \frac{-\left( d_{diff}\left( i,j \right)-o \right)}{w} \right)^{2}}$$

$$\left( 3 \right) repulsion\left( i, j, d \right)=\left\{ \begin{aligned} \left( d_{diff}\left( i,j \right)-o \right)^{2}, if d_{diff}\left( i,j \right)<o \\ 0, otherwise \end{aligned} \right.$$

$$\left( 4 \right) vdW\left( i, j, d \right)=\left( \frac{d_{opt}\left( i,j \right)}{d} \right)^{8}-2\left( \frac{d_{opt}\left( i,j \right)}{d} \right)^{4}$$

$$\left( 5 \right) electrostatic\left( i, j, d \right)=\frac{partial\_charge(i)*partial\_charge(j)}{d^{x}}$$

$$\left( 6 \right) hbond(i, j,d)=\left\{ \begin{aligned} {0, if\left( i,j \right)do not form hbond \atop1, d_{diff}\left( i, j \right)<-0.7} \\ 0, d_{diff}\left( i, j \right)\geq b \\ \frac{d_{diff}\left( i, j \right)-b}{-0.7-b}, otherwise \end{aligned} \right.$$

$$\left( 7 \right) hydrophobic(i, j,d)=\left\{ \begin{aligned} {0, not\_hydrophobic\left( i \right) or not\_hydrophobic\left( j \right) \atop1, d_{diff}\left( i, j \right)<0.5} \\ 0, d_{diff}\left( i, j \right)\geq b \\ \frac{d_{diff}\left( i, j \right)-b}{0.5-b}, otherwise \end{aligned} \right.$$

$$\left( 8 \right) non\_hydrophobic(i, j,d)=\left\{ \begin{aligned} {0, is\_hydrophobic\left( i \right) or is\_hydrophobic\left( j \right) \atop1, d_{diff}\left( i, j \right)<0.5} \\ 0, d_{diff}\left( i, j \right)\geq1.5 \\ 1.5-d_{diff}\left( i, j \right), otherwise \end{aligned} \right.$$

$$\left( 9 \right) solvation\left( i, j, d \right)=\left[ \left( solv\left( i \right)+q*partial\_charge\left( i \right) \right)volume\left( j \right)+\left( solv\left( j \right)+q*partial\_charge\left( j \right) \right)volume\left( i \right) \right]e^{-\left( \frac{d}{7.2} \right)^{2}}$$

$$\left( 10 \right) counts=num\_heavy\_atoms+ligand\_length+num\_hydrophobic\_atoms+torsions+{torsions}^{2}+\sqrt{torsions}$$

where d_opt_(i,j) = the optimal distance between atoms (the sum of the van der Waals radii); d_diff_(i,j) = d – dopt ; o offsets the optimal position of the term; w specifies the width of a Gaussian; x is the exponent of the electrostatic term; b specifies where the piecewise linear terms become zero; q determines to what extent the desolvation term is charge dependent.

**Parameters**

| term | parameters | values |
| --- | --- | --- |
| gauss(31) | O,w | (0,0.3), (0.5,0.3), (1,0.3), (1.5,0.3), (2,0.3), (2.5,0.3), (0,0.5), (1,0.5), (2,0.5), (0,0.7), (1,0.7), (2,0.7), (0,0.9), (1,0.9), (2,0.9), (3,0.9), (0,1.5), (1,1.5), (2,1.5), (3,1.5), (4,1.5), (0,2), (1,2), (2,2), (3,2), (4,2), (0,3), (1,3), (2,3), (3,3), (4,3) |
| repulsion(8) | o | 0.4, 0.2, 0, −0.2, −0.4, −0.6, −0.8, −1 |
| electrostatic(2) | x | 1,2 |
| hydrogen_bond(3) | b | 0, 0.2, 0.4 |
| hydrophobic(4) | b | 1,1.5,2,3 |
| solvation(2) | q | 0,0.01097 |

**External terms during calculation**

| Terms | No. |
| --- | --- |
| hydrogen_bond， | 6 |
| non_dir_anti_h_bond_quadratic | 3 |
| hydrogen_donor | 3 |
| hydrogen_acceptor | 3 |
| h_bond ##LJ 10-12 potential | 3 |
| num_tors_div_simple | 1 |
| num_tors_div | 1 |
| num_ligands | 1 |
| num_heavy_atoms_div | 1 |
| vdw 6-12 | 1 |

**1.4 Affiscore[6-8]**

$$\left( 1 \right) SF=Hydrophobic+Polar+Repulsive+Solvation+Entropic$$

$$\left( 2 \right)Hydrophobic=\sum_{i,j} f_{0}\left( d\left( i,j \right) \right)$$

$$\left( 3 \right)Polar=\sum_{i,j} f_{1}\left( d\left( i,j \right), i,j \right)$$

$$\left( 4 \right)Repulsive=\sum_{i,j} f_{2}\left( d\left( i,j \right), i,j \right)$$

$$\left( 5 \right)Solvation=\left( l_{5}phbe \right)+\left( l_{6}lhbe \right)$$

$$\left( 6 \right)Entropic=\left( l_{7}n\_rot \right)+\left( l_{8}\log mol weight \right)$$

$$\left( 7 \right)f_{0}\left( x \right)=l_{0}g\left( x,n_{0},n_{1} \right)+l_{1}s\left( x,n_{2}+n_{1} \right)$$

$$(8) f_{1}\left( x, i,j \right)=f_{1a}\left( x \right)f_{1b}(i,j)(1+n_{6}c_{i})\left( 1+n_{6}c_{j} \right)$$

$$\left( 9 \right)f_{2}\left( x \right)=l_{5}g(x,n_{7},n_{8})f_{1a}(x)f_{1b}(i,j)(1+n_{6}c_{i})\left( 1+n_{6}c_{j} \right)$$

$$\left( 10 \right)f_{1a}\left( x \right)=E_{HB\_distance}=l_{2}g\left( x,n_{3},n_{4} \right)+l_{3}s(x,n_{2},n_{4})$$

$$\left( 11 \right) f_{1b}\left( i,j \right)=E_{HB_{direction}}=s\left( -\left( b_{ij}v_{i} \right)\left( b_{ij}v_{j} \right) \right)-n_{5}$$

$$\left( 12 \right) E_{elec}=(1+n_{6}c_{i})\left( 1+n_{6}c_{j} \right)$$

$$\left( 13 \right) g\left( x,\mu,\sigma\right)=e^{-\frac{{(x+\mu)}^{2}}{\sigma}}$$

$$\left( 14 \right) s\left( x,\mu\right)=1/{(1+e^{10(x+\mu)})}$$

$$\left( 15 \right) d\left( i,j \right)=\sqrt{\left( x_{i}-x_{j} \right)^{2}+\left( y_{i}-y_{j} \right)^{2}+\left( z_{i}-z_{j} \right)^{2}}-r_{i}-r_{j}$$

where l_0_, l_1_, l_2_, l_3_, l_4_, l_5_, l_6_, l_7_, l_8_ are weights; Polar term includs hydrogen bonds and salt bridges and formally charged interactions; f_1a_ is a hydrogen bonds term, f_1b_ is a directionality term; vector b_lj_ is the normalized vector from atom i to atom j, v_i_ is the 'out' direction of atom i, and v_j_ is the 'in' direction of atom j; the formal charge of atom i is denoted by ci; the difference between the total number of potential protein hydrogen-bond equivalents and the actual polar interaction amount (phbe) is multiplied by l_5_, and the difference between the total number of ligand hydrogen-bond equivalents and the actual polar interaction amount (lhbe) is multiplied by l_6_.

**1.5 GalaxyDock BP2 score[9]**

$$\left( 1 \right) SF= E_{vdW,PL}+W_{1}E_{hbond,PL}+W_{2}E_{elec,PL}+W_{3}E_{sol,PL}+W_{4}E_{vdW,L}+W_{5}E_{hbond,L}+W_{6}E_{elec,L}+W_{7}E_{sol,L}+W_{8}E_{PLP\_tor}+W_{9}E_{HM}+W_{10}E_{Drugscore}$$

The first eight terms are physics-based energy terms of AutoDock4 that describe interactions between protein (P) and ligand (L) atoms (from $E_{vdW,PL}$ to $E_{sol,PL}$) and those within ligand (from $E_{vdW,L}$ to $E_{sol,L}$).

The component **E_PLP_tor_** is the ligand torsion energy term adopted from the PLP score. This term may be considered a physics-based energy term. The term considers intra-ligand torsional strains that are not accounted.

The hydrophobic matching score **E_HM_** is adopted from the X-score to describe hydrophobic effects involved in protein–ligand binding.

The last component, **E_DrugScore_**, is a knowledge-based potential that was derived in-house using the same logic as the DrugScore distance-dependent atom-pair potential.

**1.6 X-score[10]**

$$\left( 1 \right) xscore={({SF}_{HS}+{SF}_{HC}+{SF}_{HM})}/3$$

$$\left( 2 \right){SF}_{HS}=W_{0,1}+W_{1,1}VDW+W_{2,1}HB+W_{3,1}RT+W_{4,1}HS$$

$$\left( 3 \right){SF}_{Hc}=W_{0,2}+W_{1,2}VDW+W_{2,2}HB+W_{3,2}RT+W_{4,2}HC$$

$$\left( 4 \right){SF}_{HM}=W_{0,3}+W_{1,3}VDW+W_{2,3}HB+W_{3,3}RT+W_{4,3}HM$$

$$\left( 5 \right)VDW=\sum_{i,j} \left[ \left( \frac{d_{ij,0}}{d_{ij}} \right)^{8}-2\left( \frac{d_{ij,0}}{d_{ij}} \right)^{4} \right]$$

$$\left( 6 \right)HB=\sum_{i,j} f\left( d_{ij} \right)f\left( \theta_{1,ij} \right)f\left( \theta_{2,ij} \right)$$

$\left( 7 \right)RT=\sum_{i}^{ligand} {RT}_{i}$,

${RT}_{i}=\left\{ \begin{aligned} 0, if atom i is not involved in any rotor \\ 0.5, if atom i is involved in one rotor \\ 1, if atom i is involved in two rotors \\ 0.5, if atom i is involved in more than two rotors \end{aligned} \right.$

$$\left( 8 \right)HS=\sum_{i}^{ligand} {SAS}_{i}$$

$\left( 9 \right)HC=\sum_{i,j} f(d_{ij})$, $f\left( d_{ij} \right)=\left\{ \begin{aligned} 1, d_{ij}\leq d_{0}+0.5A \\ \left( 1/{1.5} \right)\left( d_{0}+2-d_{ij} \right), d_{0}+0.5A<d_{ij} \\ 0, d_{ij}>d_{0}+2.0A \end{aligned} \right.$

$$\left( 10 \right)HM=\sum_{i}^{ligand} {logP}_{i}*{HM}_{i},$$

$${HM}_{i}=\left\{ \begin{aligned} 1, hydrophobic atom i placed in hydrophobic place \\ 0, otherwise \end{aligned} \right.$$

$$\left( 11 \right) d_{0}=r_{i}+r_{j}$$

$$\left( 12 \right)f\left( d_{ij} \right)=\left\{ \begin{aligned} 1, d_{ij}\leq d_{0}-0.7A \\ \left( \frac{1}{0.7} \right)*\left( d_{0}-d_{ij} \right), \\ 0,d_{ij}>d_{0} \end{aligned} \right.d_{0}-0.7A<d_{ij}\leq d_{0}$$

$$\left( 13 \right)f\left( \theta_{1} \right)=\left\{ \begin{aligned} 1, \theta_{1}\geq120^{\circ} \\ \left( \frac{1}{60} \right)*\left( \theta_{1}-60 \right),120^{\circ}>\theta_{1}\geq60^{\circ} \\ 0,\theta_{1}<60^{\circ} \end{aligned} \right.$$

$$\left( 14 \right)f\left( \theta_{2} \right)=\left\{ \begin{aligned} 1, \theta_{2}\geq120^{\circ} \\ \left( \frac{1}{60} \right)*\left( \theta_{2}-60 \right),120^{\circ}>\theta_{2}\geq60^{\circ} \\ 0,\theta_{2}<60^{\circ} \end{aligned} \right.$$

where $d_{ij}$ is the distance between atom i and atom j; $d_{0}$ is the van der Waals distance

between the donor and the acceptor; $\theta_{1}$ and $\theta_{2}$ are the angle between hydrogen donor’s root atom, donor and acceptor, and the angle between the donor, acceptor and acceptor’s root atom; SAS represent the solvent-accessible surface.

**1.7 SMoG2016[11]**

$$\left( 1 \right)SF=KBP2016+0.535Repulsion+1.913Num\_Rotor-21.974\ln(m_{L})$$

$$\left( 2 \right)Repulsion=\sum_{ij} \frac{A_{ij}}{r_{ij}^{12}}$$

where **KBP2016** is the knowledge-based potential, **Rotor** is the number of rotatable bonds in the ligand, and **mL** is the ligand mass.

**1.8 Glide[12, 13]**

$$\left( 1 \right)SF=W_{lipo-lipo}\sum f\left( r_{lr} \right)+W_{hbond-neut-neut}\sum g\left( \Delta r \right)h\left( \Delta\alpha\right)+W_{hbond-neut-charged}\sum g\left( \Delta r \right)h\left( \Delta\alpha\right)+W_{hbond-charged-charged}\sum g\left( \Delta r \right)h\left( \Delta\alpha\right)+W_{max-metal-ion}\sum f(r_{lm})+W_{rotb}H_{rotb}+W_{polar-phob}V_{polar-phob}+W_{coul}E_{coul}+W_{vdW}E_{vdW}+solvation$$

r_lr_ is the distance between the ligand atom and the protein atom and $\Delta\alpha$ is the bond angle.

***f***, ***g***, and ***h*** are functions that give a full score (1.00) for distances or angles that lie within nominal limits and a partial score (1.00-0.00) for distances or angles that lie outside those limits but inside larger threshold values. For example, g ($\Delta r$) is 1.00 if the H…X hydrogenbond distance is within 0.25 Å of a nominal value of 1.85 Å but tails off to zero in a linear fashion if the distance lies between 2.10 and 2.50 Å. Similarly, h($\Delta\alpha$) is 1.00 if the Z-H…X angle is within 30° of 180° and decreases to zero between 150° and 120°.

The **lipophilic-lipophilic** term is defined as in Chem-Score, rewards hydrophobic interactions.

The **hydrogen-bonding** term also uses the Chem-Score form but is separated into differently weighted components that depend on whether the donor and acceptor are both neutral, one is neutral and the other is charged, or both are charged.

To include **solvation effects**, Glide 2.5 docks explicit waters into the binding site for each energetically competitive ligand pose and employs empirical scoring terms that measure the exposure of various groups to the explicit waters.

$$\left( 2 \right){SF}_{sp}=W_{1}vdW+W_{2}Coul+W_{3}Lipo+W_{4}Hbond+W_{5}Metal+W_{6}Rewards+W_{7}Rotb+W_{8}site$$

$$\left( 3 \right){SF}_{xp}=W_{1}\mathrm{LipophilicEvdW}+W_{2}\mathrm{PhobEn}+W_{3}\mathrm{PhobEnHB}+W_{4}\mathrm{PhobEnPairHB}+W_{5}\mathrm{HBond}+W_{6}\mathrm{Electro}+W_{7}\mathrm{Site}+W_{8}\pi_{\mathrm{cation}}+W_{9}\mathrm{Reward}_{\mathrm{ClBr}}+W_{10}\mathrm{LowMW}+W_{11}Penalties+W_{12}HBPenal+W_{13}ExposPenal+W_{14}\mathrm{RotB}$$

**RotB** means penalty for freezing rotable bonds

**Site** means polar interactions in the active site.

**LipophilicEvdW** means lipophilic term derived from hydrophobic grid potential at the hydrophobic ligand atoms.

**PhobEn** means hydrophobic enclosure reward.

**PhobEnPairHB** means Reward for hydrophobically packed correlated H-bonds.

**Reward_ClBr_** means reward for Cl or Br in a hydrophobic environment that pack against Asp or Glu.

**LowMW** means Reward for ligands with low molecular weight.

**Penalties** means Polar atom burial and desolvation penalties, and penalty for intra-ligand contacts.

**HBPenal** means Penalty for ligands with large hydrophobic contacts and low H-bond scores.

**ExposPenal** means Penalty for solvent-exposed ligand groups; cancels van der Waals terms.

**1.9 Chemscore[14]**

$$\left( 1 \right)SF=W_{1}E_{hbond}+W_{2}E_{metal}+W_{3}E_{hydrophobic}+W_{4}E_{entropy}+b+W_{5}E_{clash}+W_{6}E_{internal}$$

$$(2)E_{hbond}=\sum_{ij} B(r_{ij},R_{i},R_{j},\sigma_{r})B(\alpha_{ij},A_{i},A_{j},\sigma_{\alpha})B(\Delta\beta,{\Delta\beta}_{i},{\Delta\beta}_{j},\sigma_{\beta})$$

$$\left( 3 \right)E_{metal}=\sum_{mj} B\left( r_{mj},R_{1},R_{2}, \sigma_{metal} \right), R_{1}=2.2Å,R_{2}=2.6Å$$

$$\left( 4 \right)E_{hydrophobic}=\sum_{ll} B(r_{ll},R_{l1},R_{l2},\sigma_{lipo})$$

$$\left( 5 \right)\Delta E_{entropy}=1+(1-\frac{1}{N_{rot}})\sum_{r} \frac{(P_{nl}\left( r \right)-{P'}_{nl}\left( r \right))}{2}$$

$\left( 6 \right)E_{clash-hbond}=\frac{20*(r_{hbond}-r)}{\Delta G_{hbond}*r_{hbond}}, r_{hbond}=1.6Å$

$\left( 7 \right)E_{clash-metal}=\frac{20*(r_{metal}-r)}{\Delta G_{metal}*r_{metal}}, r_{metal}=1.3Å$

$\left( 8 \right)E_{clash-other}=1+\frac{4*(r_{clash}-r)}{r_{clash}}, r_{clash}=\left\{ \begin{aligned} 3.35Å for contacts to protein sulfur atoms \\ 3.10Å for all other contacts \end{aligned} \right.$

$\left( 9 \right)E_{internal}=\sum_{all rotatable bonds} A_{i}(1-\cos(n\emptyset-\emptyset_{0}))$

where $r_{ij}$ is the distance between ligand atom i and protein atom j;$\alpha_{ij}$ is the D(onor)-H…A(cceptor) angle; $r_{mj}$ is the distance between metal atom m and protein atom j;$r_{ll}$ is the the distance between protein and ligand atom for a given pair of lipophilic atoms; $R_{i},R_{j}, A_{i},A_{j}, R_{1},R_{2}, R_{l1},R_{l2},\sigma$ are constant; $\Delta\beta$ means the absolute deviation of the actual H..A-X angle from $\beta$; N_rot_ is the number of frozen rotatable bonds in the ligand; P_nl_(r) and P’_nl_(r) are the percentages of non-hydrogen atoms on either side of the rotatable bond that are not lipophilic; the parameters A, n and $\emptyset$ in the above equation are set in the ChemScore file; Internal ligand strain is accommodated by clash terms in combination with torsional strain terms of the form.

**1.10 ChemPLP[14]**

$$\left( 1 \right)SF=W_{1}f_{PLP}+W_{2}f_{lig-clash}+W_{3}f_{lig-tor}+f_{chem-tor}+W_{4}f_{chem-prot}+W_{5}f_{cons}+f_{chem-distance-hb}+f_{chem-angle-hb}+f_{chem-metal}$$

In both cases, the Piecewise Linear Potential (f_PLP_) is used to model the steric complementarity between protein and ligand, while for ChemPLP additionally the distance and angle-dependent hydrogen and metal bonding terms from ChemScore are considered (f_chem-hb_, f_chem-cho_, f_chem-met_).

Both fitness functions are capable of covalent docking (f_chem-cov_), considering flexible side chains (f_chem-prot_) and explicit water molecules as well as handling constraints (f_cons_).

**1.11 Gold score[15]**

$$\left( 1 \right)SF=W_{1}f_{pro-lig-hbond}+W_{2}f_{pro-lig-vdW}+W_{3}f_{lig-vdW}+W_{4}f_{lig-torsion}+W_{5}f_{lig-hbond}$$

$$\left( 2 \right)f_{hbond}=\sum_{DA} f(r)f(\theta)E_{pair}(DA)$$

$$\left( 3 \right)f_{vdW}=\sum_{ij} (\frac{A}{d_{ij}^{8}}-\frac{B}{d_{ij}^{4}})$$

$$\left( 4 \right)f_{torsion}=\frac{1}{2}V_{ijkl}\left[ 1+\frac{n_{ijkl}}{\left| n_{ijkl} \right|}\cos(\left| n_{ijkl} \right|*\omega_{ijkl}) \right]$$

$$\left( 5 \right)f\left( r \right)=\left\{ \begin{aligned} 1,r<0.25A \\ \frac{-1}{1.25}r,0.25\leq r\leq1.5 \\ 0,r>1.5A \end{aligned} \right.$$

$$\left( 6 \right)f\left( \theta\right)=\left\{ \begin{aligned} 1,\theta<\theta_{1} \\ \frac{-1}{\theta_{2}-\theta_{1}}\theta,\theta_{1}\leq\theta\leq\theta_{2} \\ 0,\theta>\theta_{2} \end{aligned} \right.$$

$$\left( 7 \right)E_{pair}\left( DA \right)=\left( E_{da}+E_{ww} \right)-(E_{dw}+E_{aw})$$

where r means the distance between atom donor and atom acceptor; $\theta$ is the angle between the donor, donor hydrogen atom and the plane of the lone-pairs; f_torsion_ was the torsional energy associated with four consecutively bonded atoms i, j, k, l; $\omega$ was the torsional angle; n was the periodicity; V the barrier to rotation and V are parameters taken from Clark et al; d means hydrogen bond donor and a means acceptor; w means water; the value of $E_{da},E_{ww},E_{dw} and E_{aw}$ can be got from parameter table.

**1.12 ASP[14]**

$$\left( 1 \right)SF=W_{1}E_{map}+W_{2}E_{internal-torsion}+W_{3}E_{clash}$$

$$\left( 2 \right)E_{map}=\sum_{ij} Statescore(i,j,r)$$

$$\left( 3 \right)\mathrm{Statescore}\left( i,j,r \right)=-\ln\frac{n_{obs}(i,j,r)}{n_{exp}^{ASP}(i,j,r)}$$

$$\left( 4 \right)n_{exp}^{ASP}\left( i,j,r \right)=\left\langle\frac{n_{obs}(i,j,r')}{f_{p}(i,r')f_{l}(j,r')4\pi{r'}^{2}\Delta r} \right\rangle_{r^{'}=6}^{r^{'}=8}*f_{p}\left( i,r^{'} \right)f_{l}(j,r')4\pi r^{2}\Delta r$$

$E_{internal-torsion}$ and $E_{clash}$ are from chemscore.

The reference state is the expected number of contacts if there were no interaction between the atoms (i.e. at long distances), incorporating any corrections.

Given an atom at some position the radial distribution functions (RDFs) will tell us how many other atoms we can expect to find at a distance between r to r+d_r_, where d_r_ is the bin width in the RDF and can be thought of as the ‘thickness’ of a spherical shell.

i and j are two atom types;

The average contact density is taken to be the average between 6.0 and 8.0 Å of the corrected RDF. At this long range, atoms are not considered to make any specific interactions and should ensure that the scores of the function are close to zero at this length. The two terms f_p_ and f_s_ denote the protein and ligand volume corrections to the contacts, respectively. These two terms are added to account for the difference in accessibility of different protein and ligand atoms.

**1.13 DSX[16]**

$$\left( 1 \right)SF=W_{1}{score}_{pair}+W_{2}{score}_{tors}+W_{3}{score}_{SR}$$

$$\left( 2 \right){score}_{pair}=\sum_{ij} {score}_{pair}^{DSX}(c\left( a_{i},a_{j} \right),r(a_{i},a_{j}))$$

$$\left( 3 \right){score}_{tors}=\sum_{b} \sum_{T\in b} \frac{{score}_{tors}^{DSX}(t\left( T \right),\emptyset\left( t \right))}{nT}$$

$$\left( 4 \right){score}_{SR}=\sum_{a\in P} {score}_{SR}^{DSX}(c\left( a \right),SR\left( a \right))+\sum_{a\in L} {score}_{SR}^{DSX}(c\left( a \right),SR\left( a \right))$$

$$\left( 5 \right){score}_{pair}^{DSX}\left( c\left( a_{i},a_{j} \right),r\left( a_{i},a_{j} \right) \right)=-\ln(\frac{\rho(c\left( a_{i},a_{j} \right),r\left( a_{i},a_{j} \right))}{\rho_{ref}(c\left( a_{i},a_{j} \right),r\left( a_{i},a_{j} \right))})$$

$$\left( 6 \right)\rho\left( c\left( a_{i},a_{j} \right),r\left( a_{i},a_{j} \right) \right)=\frac{\sum_{p\_l\in C} N(p,l,r)}{\Delta V(r)\sum_{p\_l\in C} \sum_{r'} {N(p,l,r')}/{\Delta V(r')}}$$

$${\left( 7 \right)\rho}_{ref}\left( r\left( a_{i},a_{j} \right) \right)=\frac{\sum_{c'} \rho\left( c',r\left( a_{i},a_{j} \right) \right)}{n_{c}}$$

$$\left( 8 \right){score}_{tors}^{DSX}\left( t\left( T \right),\emptyset\left( t \right) \right)=-\ln(\frac{\rho(t,\emptyset)}{\rho_{ref}(\emptyset)})$$

$$\left( 9 \right)\rho\left( t,\emptyset\right)=\frac{N(t,\emptyset)}{\sum_{\emptyset'} N(t,\emptyset')}$$

$${\left( 10 \right)\rho}_{ref}\left( \emptyset\right)={\sum_{t'} \rho(t',\emptyset)}/{n_{t}}$$

$$\left( 11 \right)t=t(a,b,c,d,e)$$

$$\left( 12 \right){score}_{SR}^{DSX}\left( c\left( a \right),SR\left( a \right) \right)=-\ln(\frac{\rho(c,SR)}{\rho_{ref}(c)})$$

$$\left( 13 \right)\rho\left( c,SR \right)=\frac{\sum_{l\in c} N(l,R)}{\sum_{l\in c} \sum_{SR'} N(l,SR')}$$

$${\left( 14 \right)\rho}_{ref}\left( SR \right)={\sum_{c} \rho(c,SR)}/{n_{c}}$$

$$\left( 15 \right)\mathrm{SR}\left( a_{1} \right)=\frac{SAS(l_{complexed})}{SAS(l_{uncomplexed})}=1-\frac{\Delta SAS}{{SAS}_{0}}$$

where c is a cluster type, r is the distance between atom i and atom j, n_c_ is the number of clusters, b is a central bond of a torsion T, t is a torsion type, n_T_ is the number of torsions for a given bond, SR is the SAS-ratio for a protein or ligand atom, the w_p/t/s_ are the weighting factors used, and a, b, c, d are atom types being a part of the torsion, $\emptyset$ is the actual torsion angle, e are torsion types, a_1_ is one of the ligand atoms.

**1.14 NNscore[17]**

**Features**:

1. individual term from vina 1.1.2 (gauss1, gauss2, repulsion, hydrophobic, hydrogen bond)
2. the number of receptor ligand atoms that come within 2.5 Å of each other （BINANA）
3. the energy of the electrostatic interaction between receptor ligand atoms that come within 4 Å of each other and tallies this sum by atom-type pairs. （BINANA）
4. the number of receptor ligand atoms that come within 4.0 Å of each other（BINANA）

**Net architecture**:

One single hidden layer of ten neurodes; Each neurode had a log-sigmoid activation function; The output layer consists of a single neurode corresponding to the predicted pK_d_.

**Part 2. Tables**

**Table S1**. The name and scoring functions of Formula-based feature combination

| Index | $E_{vdW}$ | $E_{hbond}$ | $E_{elec}$ | $E_{hydrophobic}$ | $E_{entropy}$ | $E_{clash}$ |
| --- | --- | --- | --- | --- | --- | --- |
| 0 | GalaxyDock BP2 | GalaxyDock BP2 | GalaxyDock BP2 | Chemsocre | SMoG2016 | Chemscore |
| 1 | Goldscore | Goldscore | NNScore | Affiniscore | Glide XP | ChemPLP |
| 2 | Smina | Smina |  | X-Score |  |  |
| 3 |  |  |  | Glide XP |  |  |

The feature combination of 010100 means a combination of the van der Waals interaction energy terms from GalaxyDock BP2 (index 0), the Hydrogen-bond interaction from Goldscore (index1), the Coulomb electrostatic energy from GalaxyDock BP2 (index 0), the Hydrophobic energy term from Affiniscore (index 1), the Entropy effect from SMoG2016 (index 0), and the Clash effect from Chemscore (index 0).

**Table S2**. The name and scoring functions of Tree-based-mean feature combination

| Index | $E_{vdW}$ | $E_{hbond}$ | $E_{elec}$ | $E_{hydrophobic}$ | $E_{entropy}$ | $E_{clash}$ |
| --- | --- | --- | --- | --- | --- | --- |
| 0 | Glide SP | ChemPLP | Autodock | Glide XP | Chemscore | Chemscore |
| 1 |  | Goldscore | Glide SP | Affiniscore | SMoG2016 | ChemPLP |
| 2 |  |  |  |  |  | ASP |

The feature combination of 000100 means a combination of the van der Waals interaction energy terms from Glide sp (index 0), the Hydrogen bond interaction from ChemPLP (index 0), the Coulomb electrostatic energy from Autodock (index 0), the Hydrophobic energy term from Affiniscore (index 1), the Entropy effect from Chemscore (index 0), and the Clash effect from Chemscore (index 0).

**Table S3**. The name and scoring functions of Tree-based-sum feature combination

| Index | $E_{vdW}$ | $E_{hbond}$ | $E_{elec}$ | $E_{hydrophobic}$ | $E_{entropy}$ | $E_{clash}$ |
| --- | --- | --- | --- | --- | --- | --- |
| 0 | Smina | Smina | NNScore | Glide XP | SMoG2016 | Chemscore |
| 1 |  |  |  | Smina | Glide XP | ChemPLP |
| 2 |  |  |  |  |  | ASP |

The feature combination of 000100 means a combination of the van der Waals interaction energy terms from Smina (index 0), the Hydrogen bond interaction from Smina (index 0), the Coulomb electrostatic energy from NNScore (index 0), the Hydrophobic energy term from Smina (index 1), the Entropy effect from SMoG2016 (index 0), and the Clash effect from Chemscore (index 0).

**Table S4**. Performance of the models built on theory-based features

| **Model Name** | **F1 score** | **Method** | **Name Reference** |
| --- | --- | --- | --- |
| 0_1_1_2_0_0 | 0.730 | Formula-based | Table S1 |
| 0_1_1_2_0_1 | 0.727 | Formula-based | Table S1 |
| 1_0_1_2_0_1 | 0.725 | Formula-based | Table S1 |
| 0_0_1_2_0_0 | 0.725 | Formula-based | Table S1 |
| 1_0_0_2_0_1 | 0.724 | Formula-based | Table S1 |
| 1_1_0_3_0_0 | 0.724 | Formula-based | Table S1 |
| 0_0_1_2_0_1 | 0.721 | Formula-based | Table S1 |
| 1_0_0_2_0_0 | 0.721 | Formula-based | Table S1 |
| 1_0_1_2_0_0 | 0.721 | Formula-based | Table S1 |
| 1_1_0_3_0_1 | 0.720 | Formula-based | Table S1 |
| 0_0_0_3_0_1 | 0.720 | Formula-based | Table S1 |
| 0_1_0_3_0_0 | 0.718 | Formula-based | Table S1 |
| 1_0_0_3_0_1 | 0.718 | Formula-based | Table S1 |
| 1_1_1_2_0_1 | 0.717 | Formula-based | Table S1 |
| 1_0_1_3_1_1 | 0.717 | Formula-based | Table S1 |
| 1_0_1_3_1_0 | 0.716 | Formula-based | Table S1 |
| 0_1_0_2_0_1 | 0.716 | Formula-based | Table S1 |
| 1_1_1_3_0_1 | 0.716 | Formula-based | Table S1 |
| 0_0_1_3_1_0 | 0.715 | Formula-based | Table S1 |
| 0_0_0_3_0_0 | 0.715 | Formula-based | Table S1 |
| 0_0_1_3_1_1 | 0.715 | Formula-based | Table S1 |
| 1_1_0_2_0_1 | 0.715 | Formula-based | Table S1 |
| 1_0_1_3_0_1 | 0.714 | Formula-based | Table S1 |
| 1_1_1_2_0_0 | 0.714 | Formula-based | Table S1 |
| 0_1_0_3_0_1 | 0.713 | Formula-based | Table S1 |
| 0_0_0_2_0_1 | 0.713 | Formula-based | Table S1 |
| 1_1_1_3_1_1 | 0.712 | Formula-based | Table S1 |
| 2_0_1_2_0_0 | 0.712 | Formula-based | Table S1 |
| 1_1_0_2_1_0 | 0.711 | Formula-based | Table S1 |
| 1_1_1_3_0_0 | 0.711 | Formula-based | Table S1 |
| 1_0_0_3_0_0 | 0.711 | Formula-based | Table S1 |
| 1_1_1_2_1_0 | 0.711 | Formula-based | Table S1 |
| 0_0_0_2_0_0 | 0.710 | Formula-based | Table S1 |
| 1_0_0_2_1_0 | 0.710 | Formula-based | Table S1 |
| 0_0_1_3_0_0 | 0.710 | Formula-based | Table S1 |
| 1_0_1_2_1_0 | 0.710 | Formula-based | Table S1 |
| 1_0_1_3_0_0 | 0.710 | Formula-based | Table S1 |
| 1_1_0_2_0_0 | 0.709 | Formula-based | Table S1 |
| 1_0_0_2_1_1 | 0.709 | Formula-based | Table S1 |
| 2_0_1_2_0_1 | 0.709 | Formula-based | Table S1 |
| 1_1_0_2_1_1 | 0.709 | Formula-based | Table S1 |
| 0_1_0_2_1_1 | 0.709 | Formula-based | Table S1 |
| 1_1_1_3_1_0 | 0.708 | Formula-based | Table S1 |
| 0_0_1_2_1_0 | 0.708 | Formula-based | Table S1 |
| 0_1_0_2_1_0 | 0.708 | Formula-based | Table S1 |
| 1_0_1_2_1_1 | 0.707 | Formula-based | Table S1 |
| 0_1_0_3_1_0 | 0.707 | Formula-based | Table S1 |
| 2_0_1_3_1_1 | 0.707 | Formula-based | Table S1 |
| 0_0_1_3_0_1 | 0.706 | Formula-based | Table S1 |
| 2_1_1_2_0_0 | 0.706 | Formula-based | Table S1 |
| 0_0_0_2_1_1 | 0.706 | Formula-based | Table S1 |
| 0_0_0_3_1_0 | 0.706 | Formula-based | Table S1 |
| 0_0_0_2_1_0 | 0.705 | Formula-based | Table S1 |
| 0_1_1_2_1_0 | 0.705 | Formula-based | Table S1 |
| 0_1_0_2_0_0 | 0.704 | Formula-based | Table S1 |
| 0_0_0_3_1_1 | 0.704 | Formula-based | Table S1 |
| 2_0_1_3_0_1 | 0.704 | Formula-based | Table S1 |
| 2_0_1_3_1_0 | 0.704 | Formula-based | Table S1 |
| 1_1_0_3_1_1 | 0.704 | Formula-based | Table S1 |
| 0_1_1_3_1_0 | 0.704 | Formula-based | Table S1 |
| 2_1_1_2_0_1 | 0.704 | Formula-based | Table S1 |
| 0_0_1_2_1_1 | 0.703 | Formula-based | Table S1 |
| 1_0_0_3_1_0 | 0.703 | Formula-based | Table S1 |
| 0_1_1_3_0_0 | 0.703 | Formula-based | Table S1 |
| 1_0_0_3_1_1 | 0.703 | Formula-based | Table S1 |
| 1_1_1_2_1_1 | 0.703 | Formula-based | Table S1 |
| 2_1_1_3_1_0 | 0.703 | Formula-based | Table S1 |
| 0_1_1_3_0_1 | 0.702 | Formula-based | Table S1 |
| 2_1_1_3_1_1 | 0.702 | Formula-based | Table S1 |
| 0_1_1_3_1_1 | 0.702 | Formula-based | Table S1 |
| 1_2_1_3_0_0 | 0.702 | Formula-based | Table S1 |
| 1_2_0_3_0_0 | 0.702 | Formula-based | Table S1 |
| 0_1_1_2_1_1 | 0.701 | Formula-based | Table S1 |
| 1_2_1_3_0_1 | 0.700 | Formula-based | Table S1 |
| 2_2_1_3_1_1 | 0.700 | Formula-based | Table S1 |
| 1_2_1_3_1_0 | 0.700 | Formula-based | Table S1 |
| 0_2_0_2_0_1 | 0.700 | Formula-based | Table S1 |
| 2_0_1_3_0_0 | 0.700 | Formula-based | Table S1 |
| 1_2_1_3_1_1 | 0.700 | Formula-based | Table S1 |
| 1_1_0_3_1_0 | 0.699 | Formula-based | Table S1 |
| 2_1_1_3_0_0 | 0.698 | Formula-based | Table S1 |
| 0_2_1_3_0_0 | 0.698 | Formula-based | Table S1 |
| 0_1_0_3_1_1 | 0.698 | Formula-based | Table S1 |
| 2_2_1_3_0_1 | 0.698 | Formula-based | Table S1 |
| 1_2_0_2_0_1 | 0.698 | Formula-based | Table S1 |
| 0_2_1_3_0_1 | 0.697 | Formula-based | Table S1 |
| 2_0_0_3_1_1 | 0.697 | Formula-based | Table S1 |
| 2_1_1_3_0_1 | 0.696 | Formula-based | Table S1 |
| 1_0_1_1_0_1 | 0.696 | Formula-based | Table S1 |
| 2_2_1_3_1_0 | 0.695 | Formula-based | Table S1 |
| 1_2_0_3_0_1 | 0.695 | Formula-based | Table S1 |
| 2_0_0_3_1_0 | 0.693 | Formula-based | Table S1 |
| 0_2_0_2_1_1 | 0.693 | Formula-based | Table S1 |
| 2_2_1_3_0_0 | 0.693 | Formula-based | Table S1 |
| 2_0_0_3_0_1 | 0.693 | Formula-based | Table S1 |
| 0_2_1_2_0_0 | 0.693 | Formula-based | Table S1 |
| 0_2_1_2_0_1 | 0.693 | Formula-based | Table S1 |
| 0_2_1_3_1_0 | 0.692 | Formula-based | Table S1 |
| 1_2_0_2_0_0 | 0.692 | Formula-based | Table S1 |
| 1_1_1_0_0_1 | 0.692 | Formula-based | Table S1 |
| 2_1_0_3_0_0 | 0.692 | Formula-based | Table S1 |
| 0_1_0_0_0_1 | 0.691 | Formula-based | Table S1 |
| 1_0_0_0_0_1 | 0.691 | Formula-based | Table S1 |
| 0_0_1_0_0_0 | 0.691 | Formula-based | Table S1 |
| 1_2_0_2_1_0 | 0.691 | Formula-based | Table S1 |
| 1_1_0_0_0_1 | 0.691 | Formula-based | Table S1 |
| 0_2_0_3_0_1 | 0.691 | Formula-based | Table S1 |
| 2_2_1_2_0_0 | 0.691 | Formula-based | Table S1 |
| 2_1_1_2_1_1 | 0.690 | Formula-based | Table S1 |
| 2_2_1_2_0_1 | 0.690 | Formula-based | Table S1 |
| 1_0_1_0_0_1 | 0.690 | Formula-based | Table S1 |
| 2_0_0_3_0_0 | 0.690 | Formula-based | Table S1 |
| 0_0_1_1_0_1 | 0.690 | Formula-based | Table S1 |
| 0_0_0_0_0_1 | 0.690 | Formula-based | Table S1 |
| 2_1_0_3_0_1 | 0.690 | Formula-based | Table S1 |
| 0_2_0_2_1_0 | 0.689 | Formula-based | Table S1 |
| 1_2_0_2_1_1 | 0.689 | Formula-based | Table S1 |
| 0_0_1_0_0_1 | 0.689 | Formula-based | Table S1 |
| 2_0_1_1_0_1 | 0.689 | Formula-based | Table S1 |
| 2_1_0_3_1_1 | 0.689 | Formula-based | Table S1 |
| 2_0_1_2_1_0 | 0.688 | Formula-based | Table S1 |
| 2_1_0_3_1_0 | 0.688 | Formula-based | Table S1 |
| 2_2_0_3_0_0 | 0.688 | Formula-based | Table S1 |
| 0_2_1_3_1_1 | 0.688 | Formula-based | Table S1 |
| 2_0_0_2_0_1 | 0.688 | Formula-based | Table S1 |
| 0_2_0_2_0_0 | 0.688 | Formula-based | Table S1 |
| 1_1_1_0_0_0 | 0.688 | Formula-based | Table S1 |
| 0_1_1_0_0_1 | 0.687 | Formula-based | Table S1 |
| 1_0_1_1_0_0 | 0.687 | Formula-based | Table S1 |
| 2_0_1_2_1_1 | 0.687 | Formula-based | Table S1 |
| 0_0_1_1_0_0 | 0.687 | Formula-based | Table S1 |
| 2_0_1_1_0_0 | 0.686 | Formula-based | Table S1 |
| 0_1_1_0_0_0 | 0.686 | Formula-based | Table S1 |
| 1_2_1_2_0_0 | 0.686 | Formula-based | Table S1 |
| 0_2_0_3_0_0 | 0.686 | Formula-based | Table S1 |
| 1_0_1_0_0_0 | 0.686 | Formula-based | Table S1 |
| 2_1_1_2_1_0 | 0.685 | Formula-based | Table S1 |
| 1_2_1_2_0_1 | 0.685 | Formula-based | Table S1 |
| 2_2_0_3_0_1 | 0.684 | Formula-based | Table S1 |
| 0_1_1_1_0_1 | 0.684 | Formula-based | Table S1 |
| 1_1_0_0_0_0 | 0.684 | Formula-based | Table S1 |
| 2_0_0_2_0_0 | 0.684 | Formula-based | Table S1 |
| 2_1_0_2_0_0 | 0.684 | Formula-based | Table S1 |
| 2_1_0_2_0_1 | 0.684 | Formula-based | Table S1 |
| 0_0_0_0_0_0 | 0.683 | Formula-based | Table S1 |
| 1_1_1_1_0_1 | 0.683 | Formula-based | Table S1 |
| 0_1_1_1_0_0 | 0.683 | Formula-based | Table S1 |
| 1_0_0_1_0_1 | 0.682 | Formula-based | Table S1 |
| 1_2_1_2_1_1 | 0.682 | Formula-based | Table S1 |
| 2_2_1_2_1_1 | 0.681 | Formula-based | Table S1 |
| 0_1_0_0_0_0 | 0.681 | Formula-based | Table S1 |
| 2_1_1_1_0_0 | 0.681 | Formula-based | Table S1 |
| 1_0_0_1_0_0 | 0.681 | Formula-based | Table S1 |
| 2_0_0_2_1_0 | 0.681 | Formula-based | Table S1 |
| 1_2_0_3_1_0 | 0.681 | Formula-based | Table S1 |
| 0_0_0_1_0_0 | 0.680 | Formula-based | Table S1 |
| 1_2_1_2_1_0 | 0.680 | Formula-based | Table S1 |
| 2_1_0_2_1_0 | 0.680 | Formula-based | Table S1 |
| 2_2_0_3_1_0 | 0.680 | Formula-based | Table S1 |
| 1_0_0_0_0_0 | 0.680 | Formula-based | Table S1 |
| 0_1_0_1_0_1 | 0.679 | Formula-based | Table S1 |
| 1_2_0_3_1_1 | 0.679 | Formula-based | Table S1 |
| 1_0_1_1_1_1 | 0.679 | Formula-based | Table S1 |
| 2_2_0_3_1_1 | 0.679 | Formula-based | Table S1 |
| 0_0_0_1_0_1 | 0.679 | Formula-based | Table S1 |
| 0_0_1_1_1_1 | 0.678 | Formula-based | Table S1 |
| 0_1_0_1_0_0 | 0.678 | Formula-based | Table S1 |
| 2_1_1_1_0_1 | 0.677 | Formula-based | Table S1 |
| 1_1_1_1_0_0 | 0.677 | Formula-based | Table S1 |
| 2_0_0_2_1_1 | 0.676 | Formula-based | Table S1 |
| 0_2_1_2_1_1 | 0.676 | Formula-based | Table S1 |
| 2_1_0_2_1_1 | 0.675 | Formula-based | Table S1 |
| 1_0_1_1_1_0 | 0.675 | Formula-based | Table S1 |
| 2_2_0_2_1_0 | 0.674 | Formula-based | Table S1 |
| 0_2_0_3_1_0 | 0.674 | Formula-based | Table S1 |
| 0_2_1_2_1_0 | 0.674 | Formula-based | Table S1 |
| 1_1_0_1_0_1 | 0.673 | Formula-based | Table S1 |
| 0_2_0_0_0_1 | 0.673 | Formula-based | Table S1 |
| 2_2_0_2_0_0 | 0.673 | Formula-based | Table S1 |
| 2_2_0_2_0_1 | 0.672 | Formula-based | Table S1 |
| 2_2_1_2_1_0 | 0.672 | Formula-based | Table S1 |
| 2_0_1_0_0_0 | 0.672 | Formula-based | Table S1 |
| 0_2_0_3_1_1 | 0.672 | Formula-based | Table S1 |
| 1_1_1_1_1_0 | 0.672 | Formula-based | Table S1 |
| 1_0_1_0_1_1 | 0.671 | Formula-based | Table S1 |
| 0_0_0_1_1_0 | 0.671 | Formula-based | Table S1 |
| 1_1_0_1_0_0 | 0.671 | Formula-based | Table S1 |
| 0_2_1_1_0_1 | 0.671 | Formula-based | Table S1 |
| 0_2_0_0_0_0 | 0.670 | Formula-based | Table S1 |
| 1_2_0_0_0_0 | 0.670 | Formula-based | Table S1 |
| 1_2_1_1_0_1 | 0.670 | Formula-based | Table S1 |
| 1_2_1_1_0_0 | 0.670 | Formula-based | Table S1 |
| 2_1_1_0_0_0 | 0.669 | Formula-based | Table S1 |
| 1_2_0_0_0_1 | 0.669 | Formula-based | Table S1 |
| 2_0_1_0_0_1 | 0.669 | Formula-based | Table S1 |
| 0_0_1_0_1_1 | 0.669 | Formula-based | Table S1 |
| 2_2_0_2_1_1 | 0.669 | Formula-based | Table S1 |
| 0_1_1_1_1_1 | 0.668 | Formula-based | Table S1 |
| 0_2_1_0_0_1 | 0.668 | Formula-based | Table S1 |
| 0_0_0_1_1_1 | 0.668 | Formula-based | Table S1 |
| 1_0_0_1_1_0 | 0.668 | Formula-based | Table S1 |
| 1_2_1_0_0_1 | 0.668 | Formula-based | Table S1 |
| 1_1_1_1_1_1 | 0.666 | Formula-based | Table S1 |
| 0_0_1_1_1_0 | 0.666 | Formula-based | Table S1 |
| 2_1_1_0_0_1 | 0.666 | Formula-based | Table S1 |
| 1_1_1_0_1_1 | 0.666 | Formula-based | Table S1 |
| 0_2_1_1_0_0 | 0.665 | Formula-based | Table S1 |
| 0_0_0_0_1_1 | 0.665 | Formula-based | Table S1 |
| 0_0_0_0_1_0 | 0.664 | Formula-based | Table S1 |
| 0_1_1_1_1_0 | 0.663 | Formula-based | Table S1 |
| 0_0_1_0_1_0 | 0.663 | Formula-based | Table S1 |
| 1_0_1_0_1_0 | 0.663 | Formula-based | Table S1 |
| 1_0_0_1_1_1 | 0.662 | Formula-based | Table S1 |
| 1_2_1_0_0_0 | 0.662 | Formula-based | Table S1 |
| 0_2_0_1_0_1 | 0.662 | Formula-based | Table S1 |
| 1_1_0_1_1_0 | 0.660 | Formula-based | Table S1 |
| 2_2_1_1_0_0 | 0.659 | Formula-based | Table S1 |
| 0_1_0_1_1_1 | 0.659 | Formula-based | Table S1 |
| 0_2_0_1_0_0 | 0.659 | Formula-based | Table S1 |
| 2_2_1_0_0_0 | 0.659 | Formula-based | Table S1 |
| 1_1_1_0_1_0 | 0.659 | Formula-based | Table S1 |
| 0_2_1_0_0_0 | 0.658 | Formula-based | Table S1 |
| 2_2_1_1_0_1 | 0.658 | Formula-based | Table S1 |
| 1_2_1_1_1_1 | 0.658 | Formula-based | Table S1 |
| 0_1_0_1_1_0 | 0.658 | Formula-based | Table S1 |
| 0_1_1_0_1_1 | 0.657 | Formula-based | Table S1 |
| 2_2_1_0_0_1 | 0.657 | Formula-based | Table S1 |
| 2_0_1_0_1_1 | 0.657 | Formula-based | Table S1 |
| 1_0_0_0_1_1 | 0.656 | Formula-based | Table S1 |
| 2_1_1_1_1_1 | 0.654 | Formula-based | Table S1 |
| 2_0_1_1_1_1 | 0.654 | Formula-based | Table S1 |
| 1_0_0_0_1_0 | 0.653 | Formula-based | Table S1 |
| 0_1_1_0_1_0 | 0.653 | Formula-based | Table S1 |
| 2_1_1_0_1_1 | 0.653 | Formula-based | Table S1 |
| 0_1_0_0_1_0 | 0.652 | Formula-based | Table S1 |
| 0_1_0_0_1_1 | 0.652 | Formula-based | Table S1 |
| 2_1_0_0_0_1 | 0.651 | Formula-based | Table S1 |
| 1_2_0_1_0_1 | 0.651 | Formula-based | Table S1 |
| 2_0_1_0_1_0 | 0.651 | Formula-based | Table S1 |
| 0_2_1_1_1_1 | 0.651 | Formula-based | Table S1 |
| 1_2_0_1_0_0 | 0.650 | Formula-based | Table S1 |
| 2_0_0_0_0_1 | 0.649 | Formula-based | Table S1 |
| 1_2_1_0_1_1 | 0.649 | Formula-based | Table S1 |
| 2_0_1_1_1_0 | 0.649 | Formula-based | Table S1 |
| 0_2_1_0_1_1 | 0.649 | Formula-based | Table S1 |
| 0_2_1_1_1_0 | 0.649 | Formula-based | Table S1 |
| 1_2_1_1_1_0 | 0.647 | Formula-based | Table S1 |
| 2_0_0_1_0_0 | 0.647 | Formula-based | Table S1 |
| 1_1_0_1_1_1 | 0.647 | Formula-based | Table S1 |
| 1_1_0_0_1_0 | 0.647 | Formula-based | Table S1 |
| 1_2_0_1_1_0 | 0.647 | Formula-based | Table S1 |
| 2_1_0_0_0_0 | 0.647 | Formula-based | Table S1 |
| 0_2_0_1_1_1 | 0.646 | Formula-based | Table S1 |
| 0_2_1_0_1_0 | 0.646 | Formula-based | Table S1 |
| 1_2_1_0_1_0 | 0.646 | Formula-based | Table S1 |
| 2_2_1_1_1_1 | 0.645 | Formula-based | Table S1 |
| 2_1_0_1_0_0 | 0.645 | Formula-based | Table S1 |
| 2_2_0_0_0_1 | 0.645 | Formula-based | Table S1 |
| 2_0_0_1_0_1 | 0.644 | Formula-based | Table S1 |
| 0_2_0_1_1_0 | 0.644 | Formula-based | Table S1 |
| 2_2_1_0_1_1 | 0.644 | Formula-based | Table S1 |
| 1_1_0_0_1_1 | 0.644 | Formula-based | Table S1 |
| 2_0_0_0_0_0 | 0.644 | Formula-based | Table S1 |
| 1_2_0_1_1_1 | 0.644 | Formula-based | Table S1 |
| 1_2_0_0_1_1 | 0.642 | Formula-based | Table S1 |
| 2_1_0_1_0_1 | 0.641 | Formula-based | Table S1 |
| 2_1_1_1_1_0 | 0.641 | Formula-based | Table S1 |
| 2_1_1_0_1_0 | 0.641 | Formula-based | Table S1 |
| 2_2_1_1_1_0 | 0.637 | Formula-based | Table S1 |
| 2_1_0_1_1_0 | 0.636 | Formula-based | Table S1 |
| 2_2_1_0_1_0 | 0.635 | Formula-based | Table S1 |
| 0_2_0_0_1_0 | 0.635 | Formula-based | Table S1 |
| 1_2_0_0_1_0 | 0.634 | Formula-based | Table S1 |
| 2_2_0_1_0_0 | 0.634 | Formula-based | Table S1 |
| 2_0_0_1_1_1 | 0.633 | Formula-based | Table S1 |
| 0_2_0_0_1_1 | 0.633 | Formula-based | Table S1 |
| 2_2_0_1_0_1 | 0.633 | Formula-based | Table S1 |
| 2_0_0_0_1_0 | 0.633 | Formula-based | Table S1 |
| 2_0_0_1_1_0 | 0.633 | Formula-based | Table S1 |
| 2_2_0_1_1_0 | 0.632 | Formula-based | Table S1 |
| 2_1_0_1_1_1 | 0.632 | Formula-based | Table S1 |
| 2_2_0_0_0_0 | 0.631 | Formula-based | Table S1 |
| 2_1_0_0_1_0 | 0.630 | Formula-based | Table S1 |
| 2_0_0_0_1_1 | 0.630 | Formula-based | Table S1 |
| 2_2_0_1_1_1 | 0.629 | Formula-based | Table S1 |
| 2_2_0_0_1_0 | 0.625 | Formula-based | Table S1 |
| 2_2_0_0_1_1 | 0.625 | Formula-based | Table S1 |
| 2_1_0_0_1_1 | 0.624 | Formula-based | Table S1 |
| 0_0_0_0_1_2 | 0.655 | Tree-based-mean | Table S2 |
| 0_0_1_0_0_1 | 0.648 | Tree-based-mean | Table S2 |
| 0_0_1_0_0_0 | 0.646 | Tree-based-mean | Table S2 |
| 0_0_1_0_0_2 | 0.643 | Tree-based-mean | Table S2 |
| 0_0_0_0_1_1 | 0.640 | Tree-based-mean | Table S2 |
| 0_0_1_0_1_1 | 0.639 | Tree-based-mean | Table S2 |
| 0_0_1_0_1_2 | 0.636 | Tree-based-mean | Table S2 |
| 0_0_1_0_1_0 | 0.635 | Tree-based-mean | Table S2 |
| 0_0_0_0_0_0 | 0.634 | Tree-based-mean | Table S2 |
| 0_0_0_0_0_1 | 0.632 | Tree-based-mean | Table S2 |
| 0_0_0_0_0_2 | 0.632 | Tree-based-mean | Table S2 |
| 0_0_0_0_1_0 | 0.630 | Tree-based-mean | Table S2 |
| 0_0_1_1_1_1 | 0.601 | Tree-based-mean | Table S2 |
| 0_0_1_1_0_2 | 0.599 | Tree-based-mean | Table S2 |
| 0_0_1_1_1_0 | 0.598 | Tree-based-mean | Table S2 |
| 0_0_1_1_1_2 | 0.597 | Tree-based-mean | Table S2 |
| 0_0_1_1_0_0 | 0.597 | Tree-based-mean | Table S2 |
| 0_0_1_1_0_1 | 0.597 | Tree-based-mean | Table S2 |
| 0_0_0_1_0_2 | 0.586 | Tree-based-mean | Table S2 |
| 0_0_0_1_1_2 | 0.586 | Tree-based-mean | Table S2 |
| 0_0_0_1_1_0 | 0.585 | Tree-based-mean | Table S2 |
| 0_0_0_1_0_0 | 0.584 | Tree-based-mean | Table S2 |
| 0_0_0_1_1_1 | 0.576 | Tree-based-mean | Table S2 |
| 0_0_0_1_0_1 | 0.570 | Tree-based-mean | Table S2 |
| 0_0_0_0_1_1 | 0.700 | Tree-based-sum | Table S3 |
| 0_0_0_0_0_1 | 0.698 | Tree-based-sum | Table S3 |
| 0_0_0_0_1_2 | 0.698 | Tree-based-sum | Table S3 |
| 0_0_0_0_1_0 | 0.695 | Tree-based-sum | Table S3 |
| 0_0_0_0_0_0 | 0.693 | Tree-based-sum | Table S3 |
| 0_0_0_0_0_2 | 0.690 | Tree-based-sum | Table S3 |
| 0_0_0_1_0_0 | 0.660 | Tree-based-sum | Table S3 |
| 0_0_0_1_0_2 | 0.657 | Tree-based-sum | Table S3 |
| 0_0_0_1_0_1 | 0.652 | Tree-based-sum | Table S3 |
| 0_0_0_1_1_1 | 0.649 | Tree-based-sum | Table S3 |
| 0_0_0_1_1_0 | 0.647 | Tree-based-sum | Table S3 |
| 0_0_0_1_1_2 | 0.647 | Tree-based-sum | Table S3 |

**Part 3. Figures**


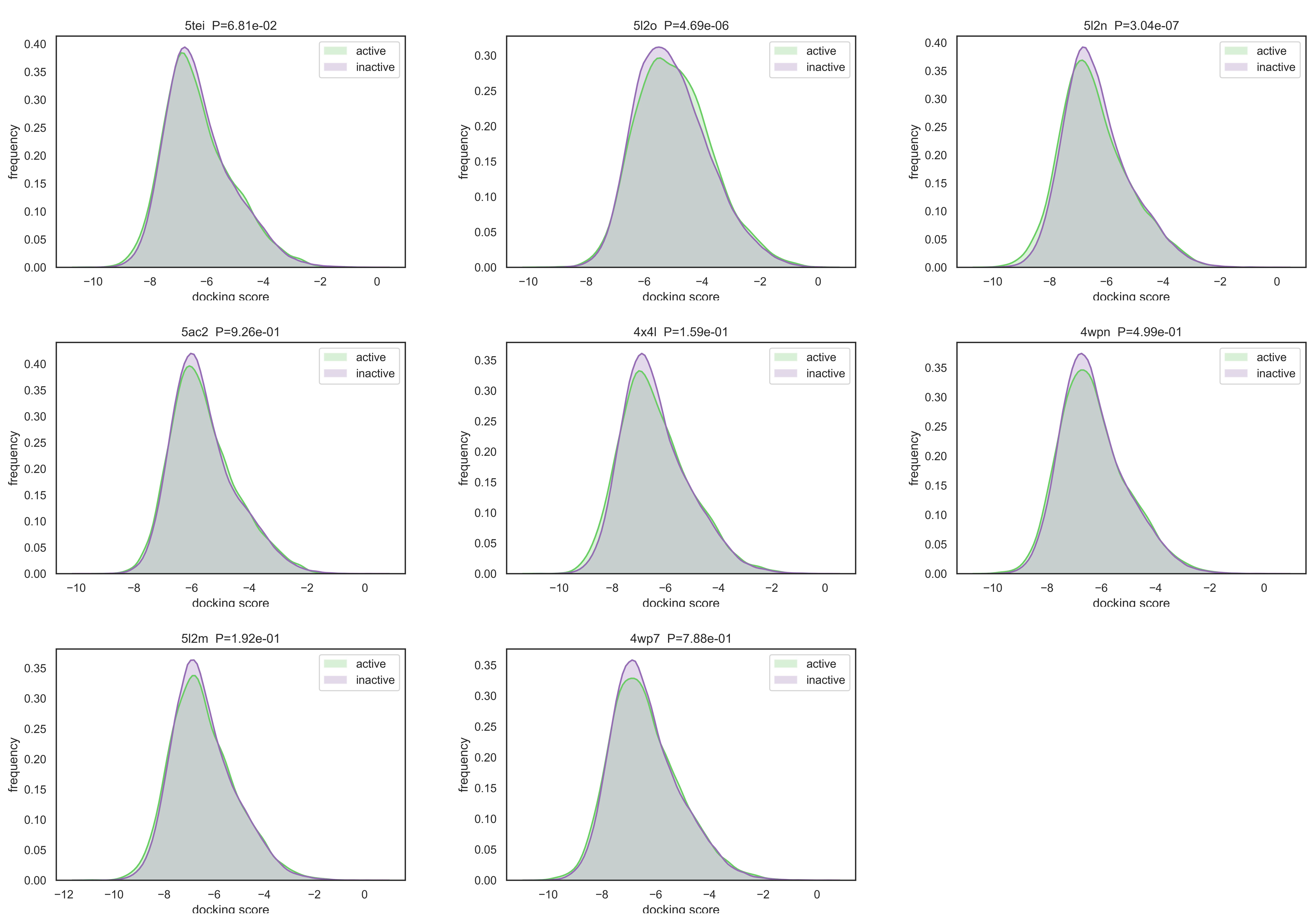


**(A)**


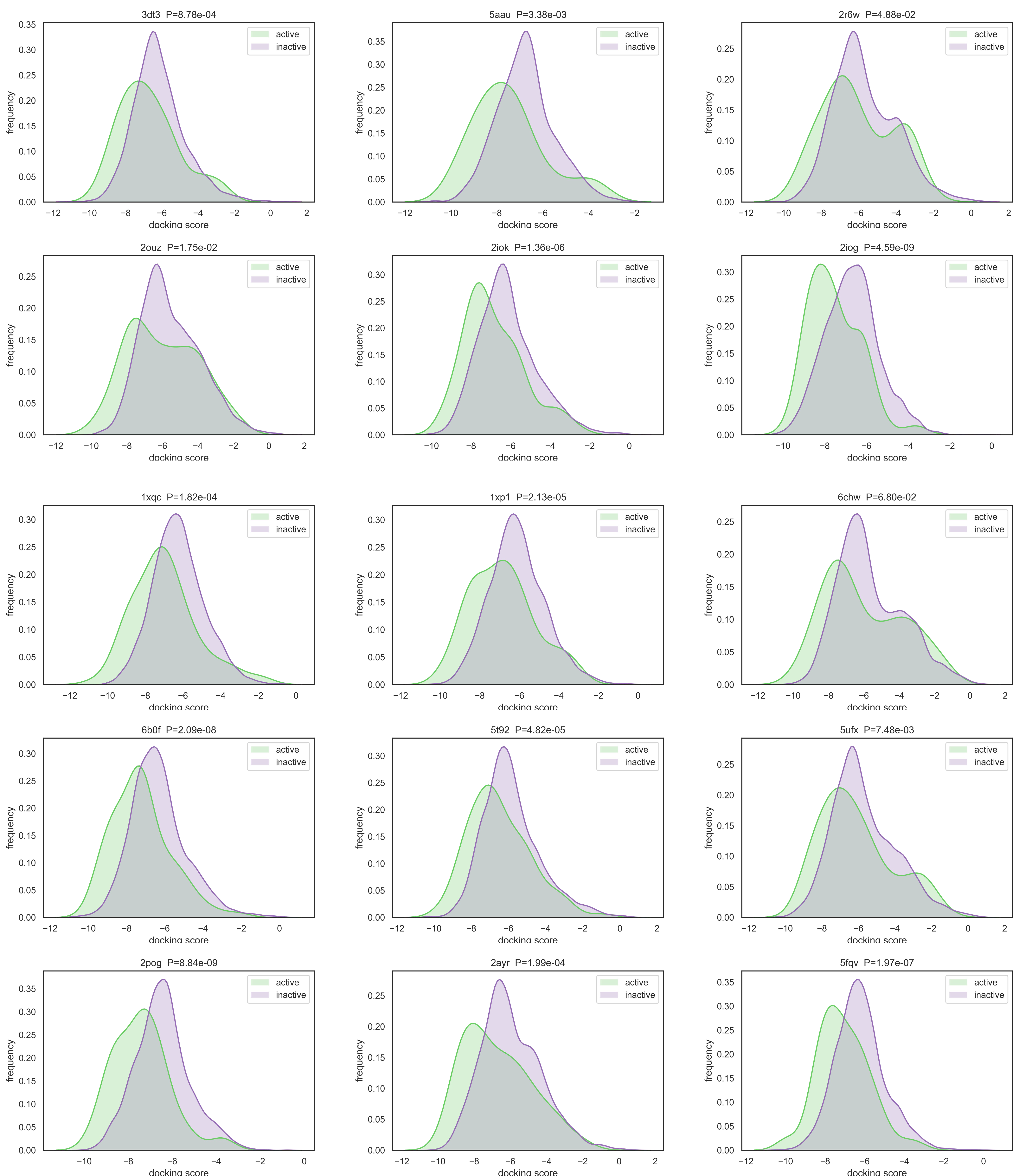


**(B)**

**
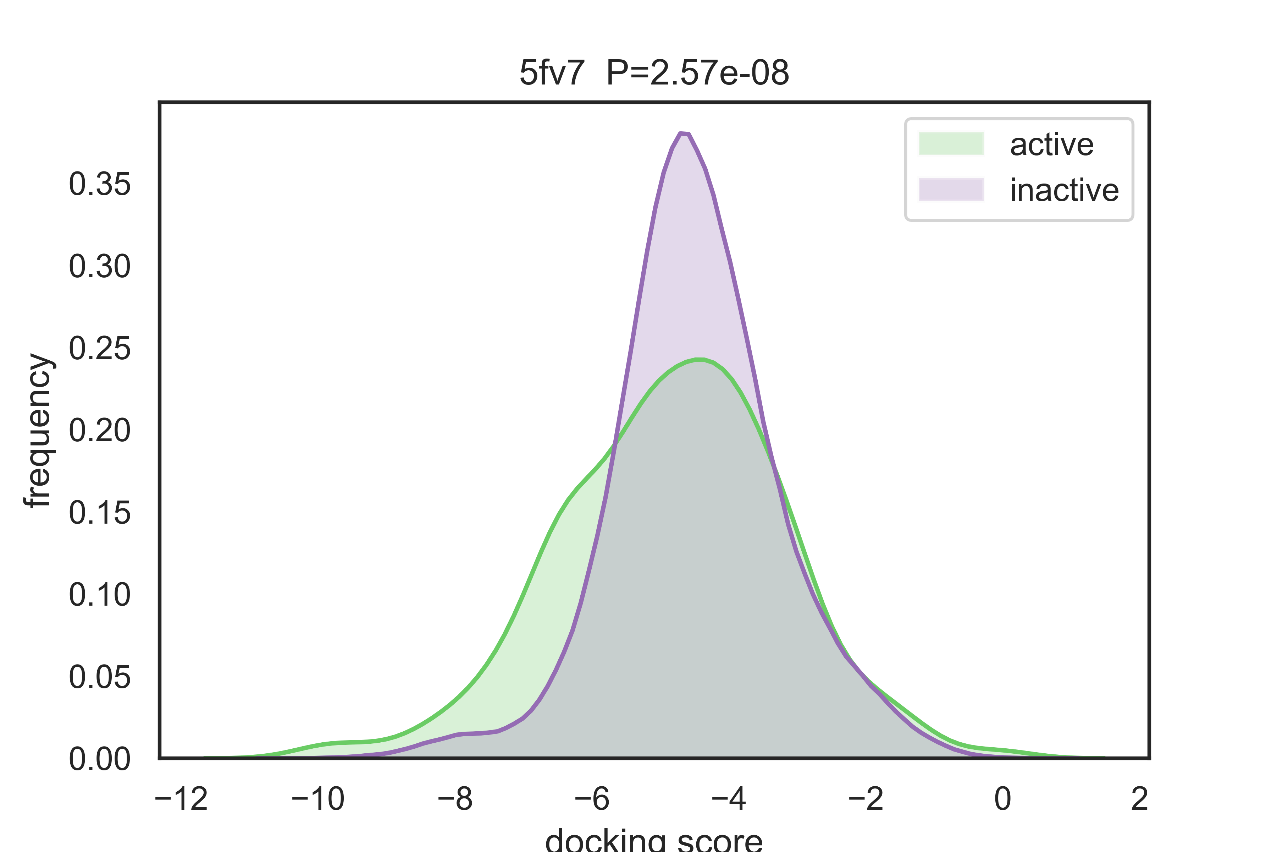
**

**(C)**

**
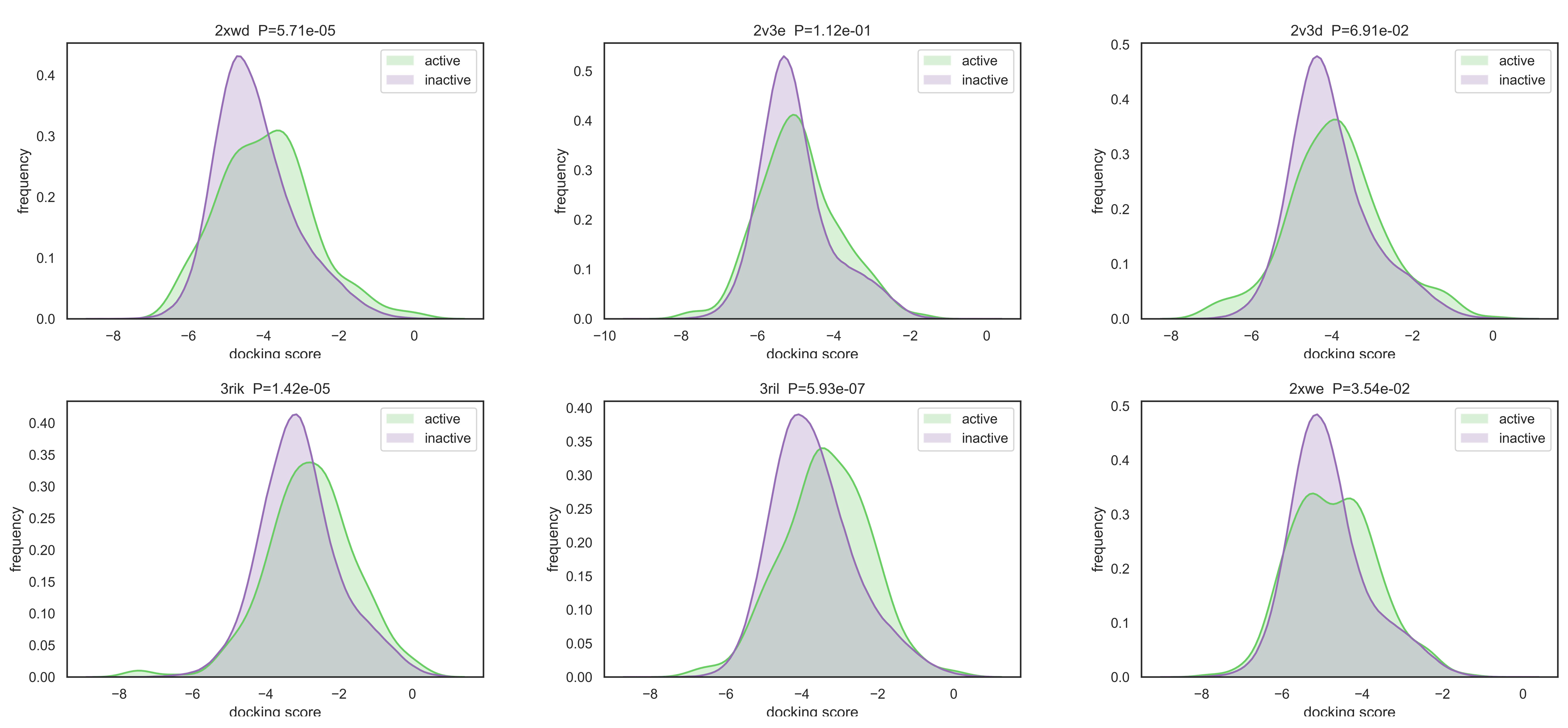
**

**(D)**

**
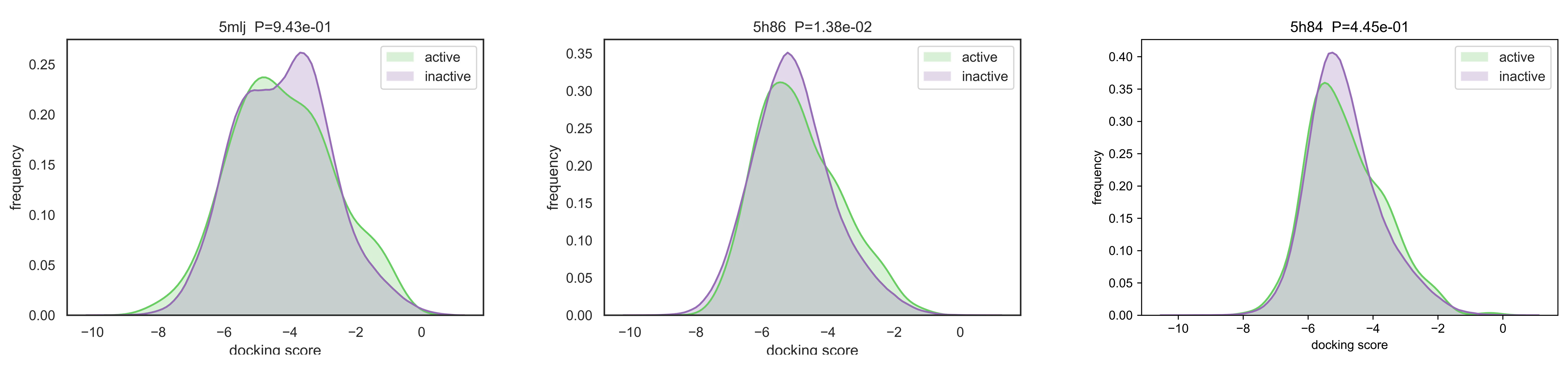
**

**(E)**

**
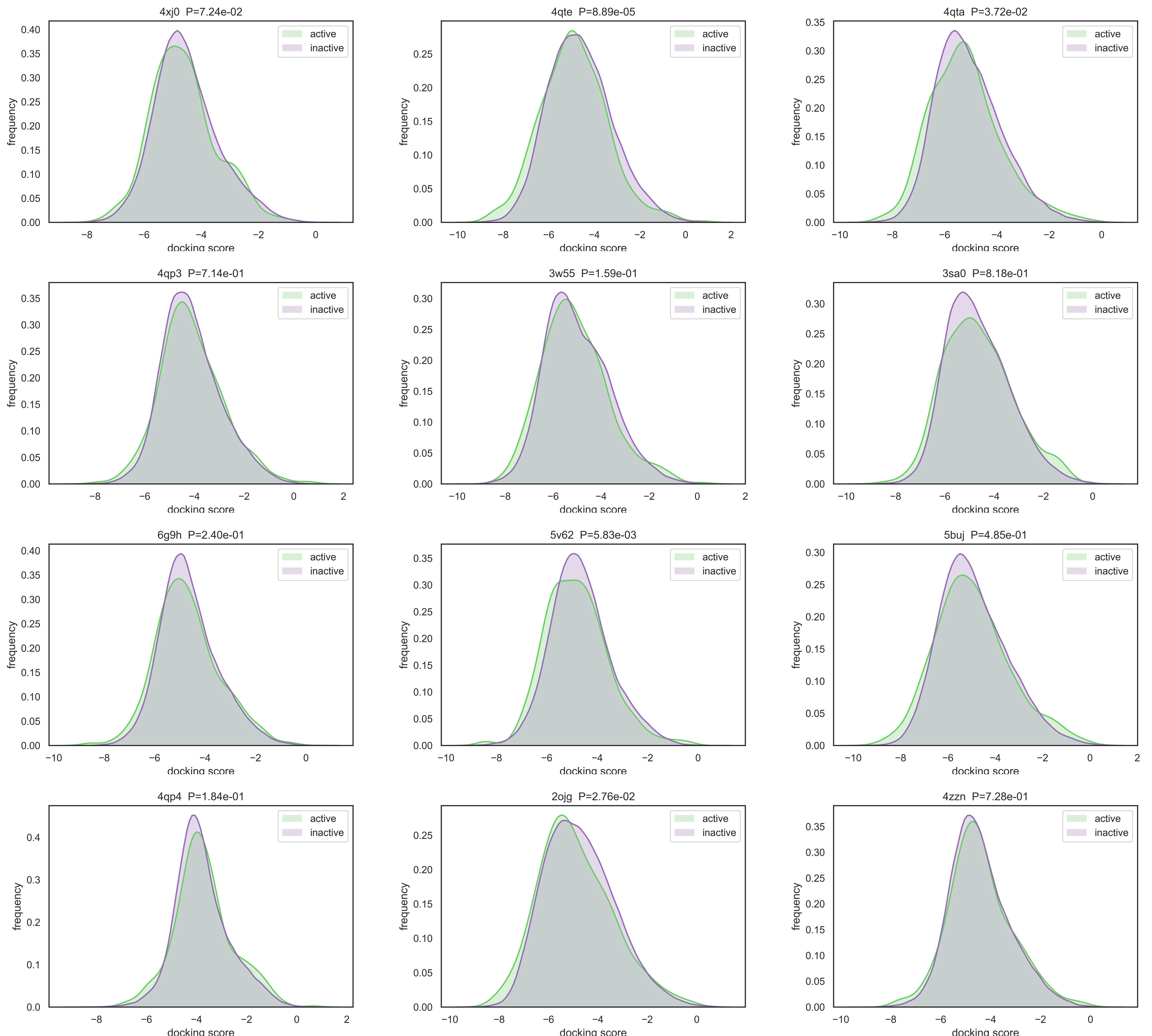
**

**(F)**

**
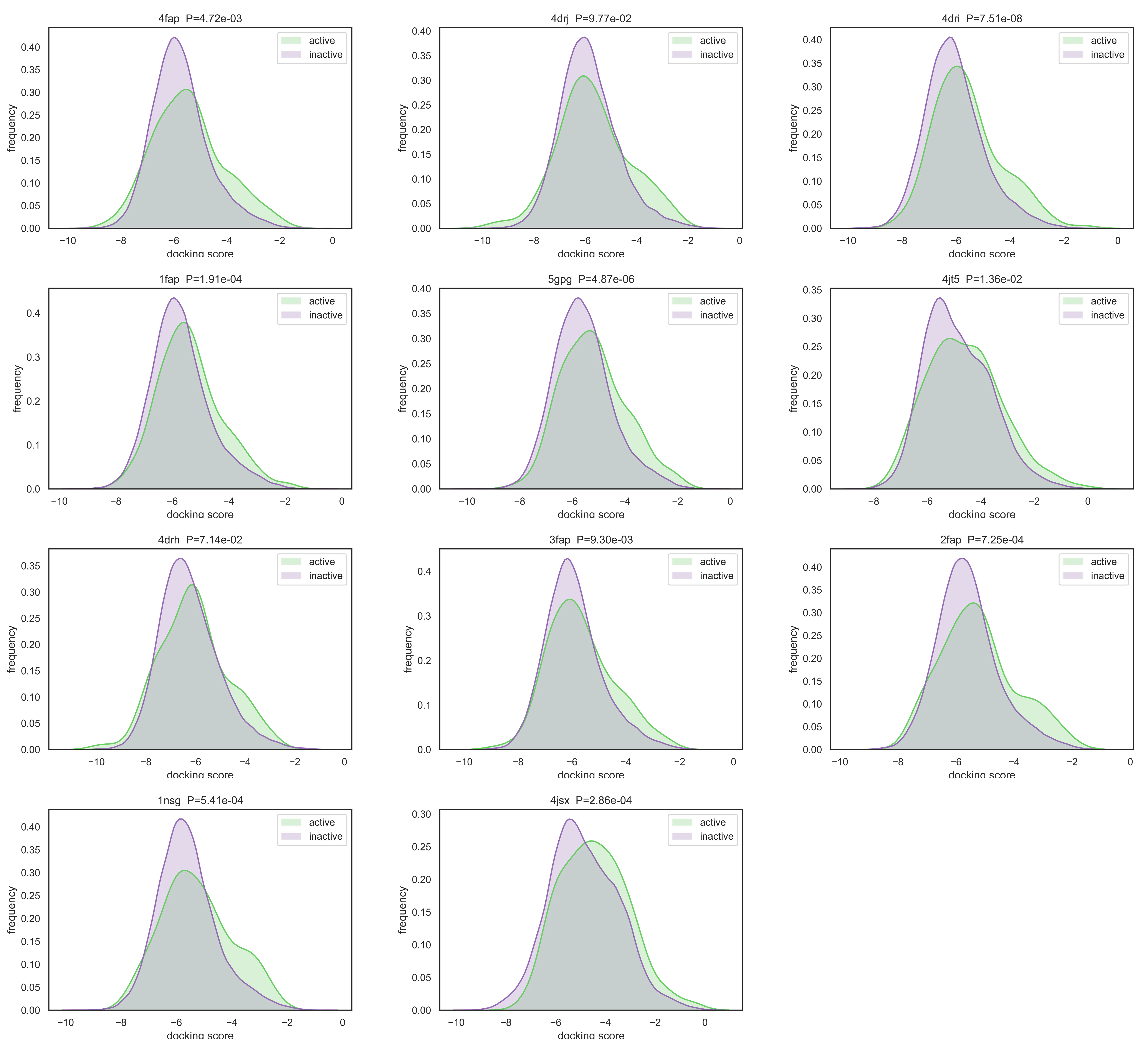
**

**(G)**

**
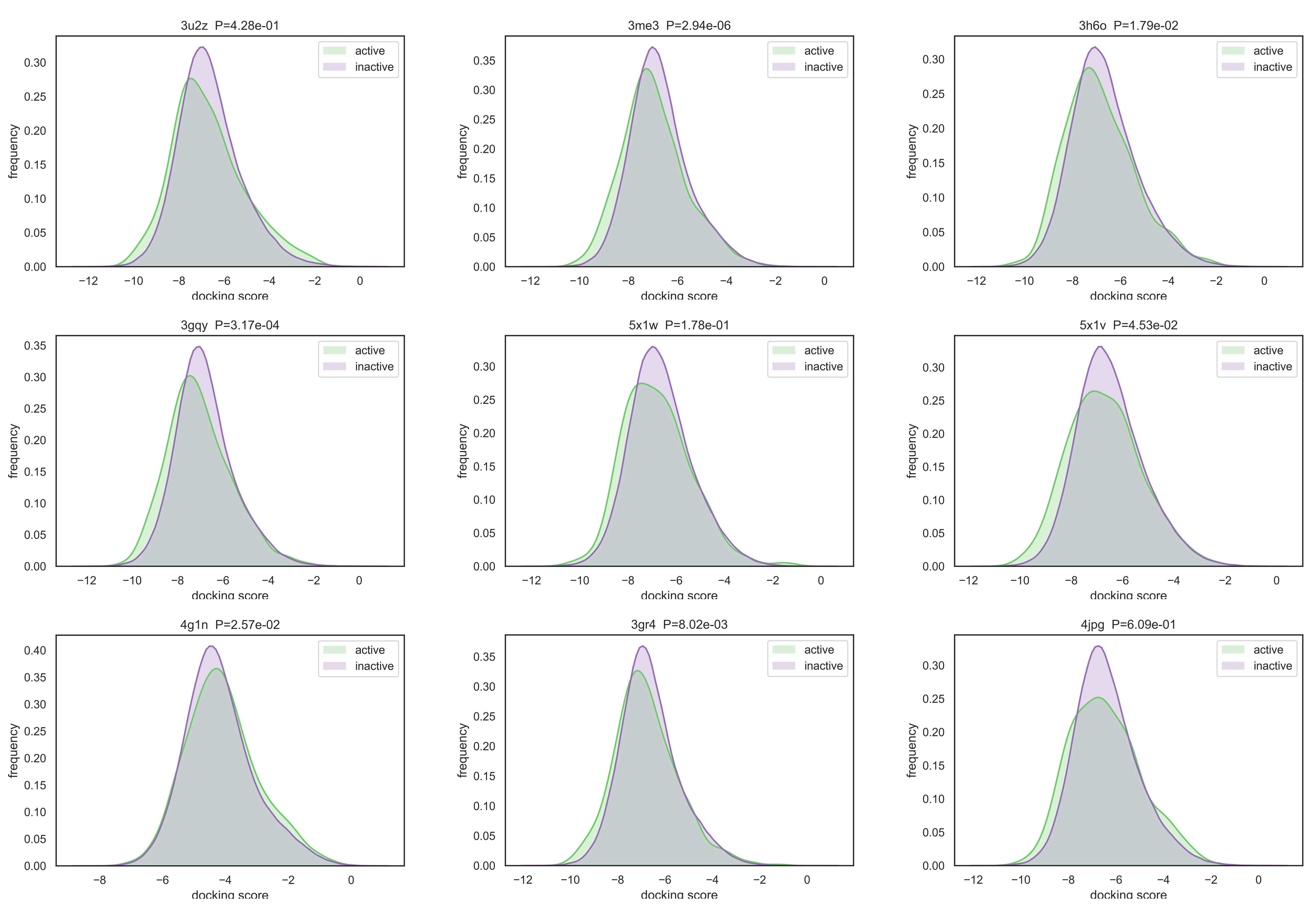
**

**(H)**

**
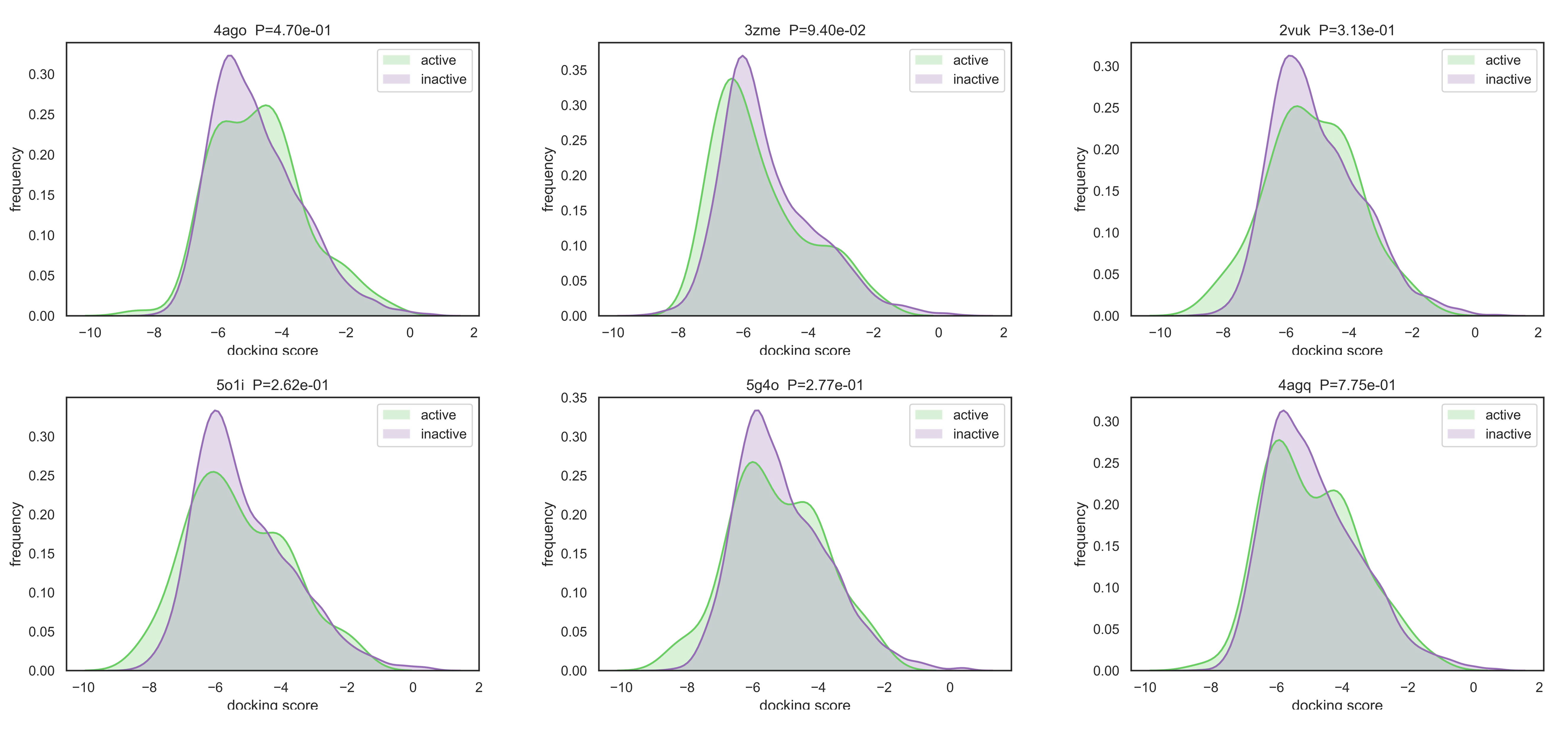
**

**(I)**

**
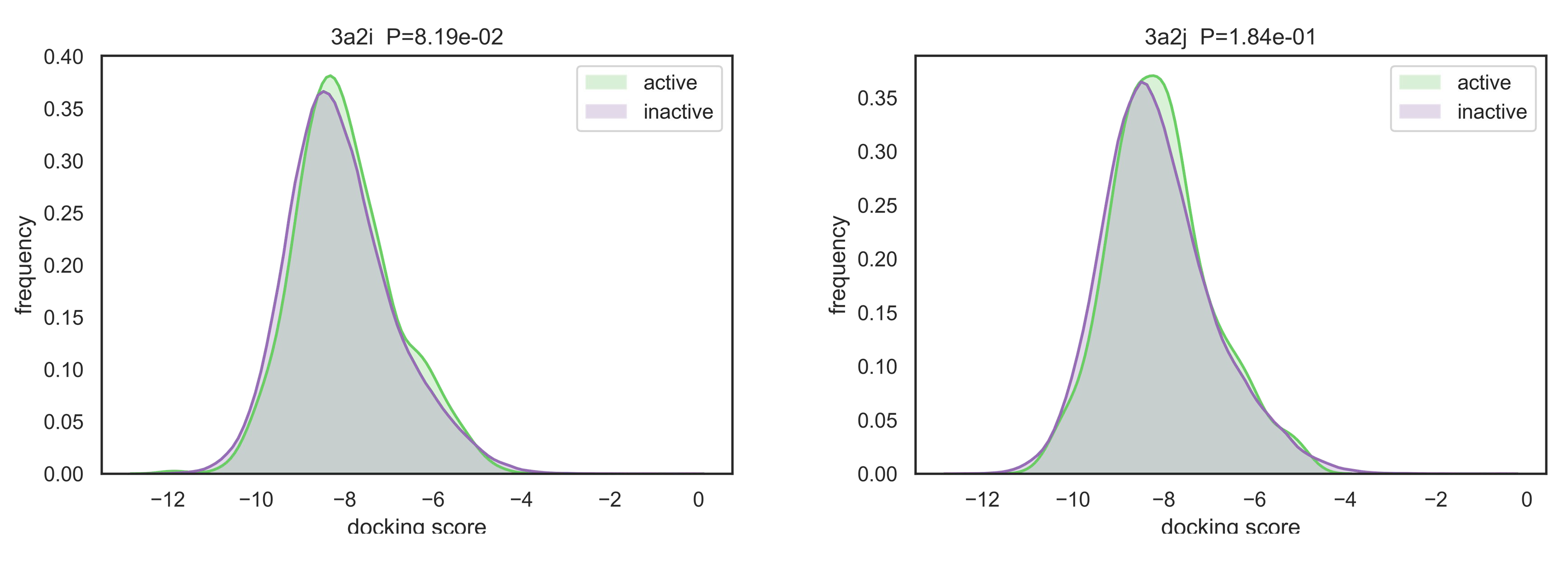
**

**(J)**

**Figure S1.** The frequency distribution plots of docking score of different pdb structures for targets (A) ALDH1, (B) ESR1_ant, (C) FEN1, (D) GBA, (E) KAT2A, (F) MAPK1, (G) MTORC1, (H) PKM2, (I) TP53 and (J) VDR.



**(A)**

**

(B)**

**

(C)**

**

(D)**

**

(E)**

**

(F)**

**Figure S2.** The importance of various scoring functions for different interactions (A) Van der Waals interaction, (B) Hydrogen bond interaction, (C) Coulomb potential, (D) Hydrophobic energy term, (E) Entropy effect, and (F) Clash effect.

**

(A)**

**

(B)**

**Figure S3.** The impact of feature-length on model performance. A): the change of the model performance after feature-length reduction, B): the change of the model performance after feature-length increase.

**Reference**

1. Morris GM, Goodsell DS, Halliday RS et al. Automated docking using a Lamarckian genetic algorithm and an empirical binding free energy function, Journal of Computational Chemistry 1998;19:1639-1662.

2. Morris GM, Goodsell DS, Huey R et al. Distributed automated docking of flexible ligands to proteins: Parallel applications of AutoDock 2.4, Journal of Computer-Aided Molecular Design 1996;10:293-304.

3. Huey R, Morris GM, Olson AJ et al. A semiempirical free energy force field with charge-based desolvation, Journal of Computational Chemistry 2007;28:1145-1152.

4. Trott O, Olson AJ. Software News and Update AutoDock Vina: Improving the Speed and Accuracy of Docking with a New Scoring Function, Efficient Optimization, and Multithreading, Journal of Computational Chemistry 2010;31:455-461.

5. Koes DR, Baumgartner MP, Camacho CJ. Lessons Learned in Empirical Scoring with smina from the CSAR 2011 Benchmarking Exercise, Journal of Chemical Information and Modeling 2013;53:1893-1904.

6. Schnecke V, Kuhn LA. Virtual screening with solvation and ligand-induced complementarity. In: Perspectives in Drug Discovery and Design. 2000, p. 171-190.

7. Zavodszky MI, Sanschagrin PC, Kuhn LA et al. Distilling the essential features of a protein surface for improving protein-ligand docking, scoring, and virtual screening, Journal of Computer-Aided Molecular Design 2002;16:883-902.

8. Zavodszky MI, Kuhn LA. Side-chain flexibility in protein–ligand binding: The minimal rotation hypothesis, Protein Science 2005;14:1104-1114.

9. Baek M, Shin W-H, Chung HW et al. GalaxyDock BP2 score: a hybrid scoring function for accurate protein-ligand docking, Journal of Computer-Aided Molecular Design 2017;31:653-666.

10. Wang RX, Lai LH, Wang SM. Further development and validation of empirical scoring functions for structure-based binding affinity prediction, Journal of Computer-Aided Molecular Design 2002;16:11-26.

11. Debroise T, Shakhnovich EI, Cheron N. A Hybrid Knowledge-Based and Empirical Scoring Function for Protein-Ligand Interaction: SMoG2016, Journal of Chemical Information and Modeling 2017;57:584-593.

12. Friesner RA, Banks JL, Murphy RB et al. Glide: A new approach for rapid, accurate docking and scoring. 1. Method and assessment of docking accuracy, Journal of Medicinal Chemistry 2004;47:1739-1749.

13. Glide user guide.

14. Gold user guide.

15. Jones G, Willett P, Glen RC et al. Development and validation of a genetic algorithm for flexible docking11Edited by F. E. Cohen, Journal of Molecular Biology 1997;267:727-748.

16. Neudert G, Klebe G. DSX: A Knowledge-Based Scoring Function for the Assessment of Protein-Ligand Complexes, Journal of Chemical Information and Modeling 2011;51:2731-2745.

17. Durrant JD, McCammon JA. NNScore 2.0: A Neural-Network Receptor-Ligand Scoring Function, Journal of Chemical Information and Modeling 2011;51:2897-2903.
